# IKK2/NF-κB Activation in Astrocytes Reduces amyloid β Deposition: A Process Associated with Specific Microglia Polarization

**DOI:** 10.3390/cells10102669

**Published:** 2021-10-06

**Authors:** Shu Yang, Alexander Magnutzki, Najwa Ouali Alami, Michael Lattke, Tabea Melissa Hein, Judith Stefanie Scheller, Carsten Kröger, Franz Oswald, Deniz Yilmazer-Hanke, Thomas Wirth, Bernd Baumann

**Affiliations:** 1 Institute of Physiological Chemistry, Ulm University, Albert-Einstein-Allee 11, 89081 Ulm, Germany; yang.shu@uni-ulm.de (S.Y.); alexander.magnutzki@adsi.ac.at (A.M.); tabea.hein@uni-ulm.de (T.M.H.); judith.scheller@uni-ulm.de (J.S.S.); kroeger.carsten@googlemail.com (C.K.); 2 Institute of Clinical Neuroanatomy, Ulm University, Helmholtzstraße 8/1, 89081 Ulm, Germany; najwa.ouali@uni-ulm.de (N.O.A.); deniz.yilmazer-hanke@uni-ulm.de (D.Y.-H.); 3 Neural Stem Cell Biology Laboratory, The Francis Crick Institute, London NW1 1AT, UK; michael.lattke@crick.ac.uk; 4 Center for Internal Medicine, Department of Internal Medicine I, University Medical Center Ulm, 89081 Ulm, Germany; franz.oswald@uni-ulm.de

**Keywords:** neuroinflammation, Alzheimer’s disease, NF-κB, IKK, astrocytes, astrogliosis, microgliosis, microglia polarization, Aβ, plaque clearance

## Abstract

Alzheimer’s disease (AD) is a common neurodegenerative disease that is accompanied by pronounced neuroinflammatory responses mainly characterized by marked microgliosis and astrogliosis. However, it remains open as to how different aspects of astrocytic and microglial activation affect disease progression. Previously, we found that microglia expansion in the spinal cord, initiated by IKK2/NF-κB activation in astrocytes, exhibits stage-dependent beneficial effects on the progression of amyotrophic lateral sclerosis. Here, we investigated the impact of NF-κB-initiated neuroinflammation on AD pathogenesis using the APP23 mouse model of AD in combination with conditional activation of IKK2/NF-κB signaling in astrocytes. We show that NF-κB activation in astrocytes triggers a distinct neuroinflammatory response characterized by striking astrogliosis as well as prominent microglial reactivity. Immunohistochemistry and Congo red staining revealed an overall reduction in the size and number of amyloid plaques in the cerebral cortex and hippocampus. Interestingly, isolated primary astrocytes and microglia cells exhibit specific marker gene profiles which, in the case of microglia, point to an enhanced plaque clearance capacity. In contrast, direct IKK2/NF-κB activation in microglia results in a pro-inflammatory polarization program. Our findings suggest that IKK2/NF-κB signaling in astrocytes may activate paracrine mechanisms acting on microglia function but also on APP processing in neurons.

## 1. Introduction

The pathogenesis of Alzheimer’s disease (AD) is not only restricted to the neuronal compartment, but is also strongly interconnected with neuroinflammatory mechanisms in the brain. Parenchymal amyloid-beta (Aβ) accumulation and protein aggregation trigger an innate immune response mediated by microglia and astroglia, which in turn contributes to disease progression and cognitive decline owing to glial activation and upregulation of inflammatory mediators like IL-1β, IL-6, and TNF-α. Neuroinflammation is not only a secondary event induced by emerging senile plaques and/or by intraneuronal neurofibrillary tangles (NFTs)-formed tau aggregates, but actively contributes to AD pathogenesis. Astrocytes and microglia are preferentially activated in the close vicinity of plaques, thereby promoting the secretion of diverse pro-inflammatory mediators including cytokines and components of the complement system. This creates a neuroinflammatory condition able to trigger neurodegenerative processes. At the same time, the phagocytic activity of microglia cells fosters Aβ clearance demonstrating a neuroprotective role of the immune response [1,2,3,4,5].

The NF-κB family of transcription factors consists of five members named RelA (p65), RelB, c-Rel, p50/p105, and p52/p100, which form various homo- and hetero-dimers. In resting cells, nuclear translocation of NF-κB dimers is prevented by the interaction with an inhibitor of κB (IκB) proteins. Upon inflammation, the activated IκB kinase complex (IKK) phosphorylates IκB proteins and marks them for proteasomal degradation. IKK2 is the pivotal kinase in canonical NF-κB signaling and an essential regulator of inflammatory responses [6]. The IKK/NF-κB signaling system is already active during neuroectodermal development [7], is present in the different cell types of the adult nervous system [8] and its activation in astrocytes and microglia, for example, Aβ-mediated toll-like receptor signaling is a critical step in the initiation of neuroinflammation in AD [9,10].

An important mechanism to prevent pathological Aβ-deposition is Aβ-clearance via phagocytosis. In AD, microglial cells differentiate into a phagocytosis-proficient phenotype near amyloid plaques and are able to clear pathological Aβ deposits from the neuropil [11,12]. Microglia, often referred to as resident macrophages of the brain, are highly plastic cells, which are able to change from a homeostatic surveillance state to reactive phenotypes depending on the demands/insults of their environment [13]. Although previous studies on post-mortem material of AD patients treated with immunotherapy against Aβ42 showed that the activation of anti-inflammatory and pro-inflammatory pathways co-exist simultaneously [14,15], cytokine release was slightly more prone to create an anti-inflammatory condition when compared to unimmunized AD patients.

Microglia polarization phenotypes have been categorized in analogy to macrophages and to simplify data interpretation into M1-like (classically activated, pro-inflammatory) and M2-like (alternatively activated, anti-inflammatory). However, several recent studies provided clear evidence that many more functional microglia populations exist beyond M1 and M2 [16,17,18]. Nevertheless, M2-like-polarized microglia are well known for their increased capability of Aβ uptake and clearance by phagocytosis [19]. Microglia polarization triggered by environmental factors is accompanied by changes in their motility, which represents an essential characteristic of microglia cells undergoing a new functional specification. Microglial motility changes in AD and microglial clusters have been identified around Aβ plaques, indicating a movement of microglia toward Aβ aggregates [20]. In addition, the impairment of directed motility was associated with decreased phagocytic activity and was observed together with morphological changes in plaque-associated cells in AD mouse models [21]. Moreover, Aβ-immunotherapy clinical studies shed light on the controversial effect of the immune response in AD and aging by showing a markedly reduced plaque load associated with microglial changes toward a phagocytic phenotype characterized by high expression levels of the activation marker, CD68 [22]. These cells display small, round, granular structures within the cytoplasm, consistent with the expected appearance of lysosomes within phagocytic microglia in contrast with the surveillant ramified microglia-like shapes of the healthy brain.

Independent of Aβ-phagocytosis, proteolytic processing of the amyloid precursor protein (APP) and Aβ biogenesis are also critical factors determining overall plaque formation. APP is a transmembrane glycoprotein with a large luminal domain and a short cytoplasmic domain that can be processed through amyloidogenic or non-amyloidogenic pathways. The non-amyloidogenic pathway is an innate way to prevent the generation of Aβ. In this pathway, APP is processed initially by the α-secretase ADAM10 within the Aβ domain, generating the soluble α-APP fragments (sAPPα) and the C-terminal fragment α (CTFα, C83). C83 is then cleaved by γ-secretase, producing non-toxic P3 and AICD fragments. The β-secretase BACE2 can also cleave APP at the α-secretase site preventing the generation of Aβ. However, when APP is first cleaved by the β-secretase BACE1, the amyloidogenic pathway takes place with successive cleavage of APP by the γ-secretase, leading to increased Aβ production [23].

In previous work, we addressed the role of astrocytic IKK2/NF-κB activation in amyotrophic lateral sclerosis (ALS) pathogenesis using the tetracycline-regulated expression of constitutively active IKK2 (IKK2-CA) under the control of the GFAP promoter (GFAP.tTA/tetO.IKK2-CA) [24,25]. Interestingly, IKK2/NF-κB activation in astrocytes induces an initial protective phenotype in pre-symptomatic stages of ALS, which was associated with the expansion of beneficially acting microglia in the spinal cord as well as decreased motoneuron degeneration and overall delayed ALS disease onset [25].

Given the different functional states of microglia, which are active in the course of AD [17,26,27], we investigated whether astrocytic IKK2/NF-κB activation and its effects on microglia homeostasis are able to affect Aβ pathology in the APP23 mouse model of AD [28]. For this purpose, we established a novel triple transgenic AD mouse model (Ttg) carrying the Swedish mutation (APP23) together with conditional neuroinflammation, restricted to astrocytes (GFAP.tTA/tetO.IKK2-CA), in order to dissect the consequences of the astrocyte-driven inflammatory response on microglial cells and AD plaque pathology. Ttg mice and control littermates were assessed by protein analyses techniques, gene expression profiling, flow-cytometry, and immunofluorescence stainings of different brain regions in 12-month-old male mice.

Our findings demonstrate that astrocytic NF-κB-mediated neuroinflammation (i) reduced plaque number and amyloid burden in different brain regions (cerebral cortex, hippocampus, and entorhinal cortex), (ii) triggered a significant increase in ADAM17 and BACE2 gene expression in the hippocampus, possibly shifting APP processing toward the non-amyloidogenic pathway, and (iii) amplified microglia cells, exhibiting a polarization status facilitating Aβ-clearance.

## 2. Materials and Methods

### 2.1. Transgenic Mice

First, we crossed B6.Cg-Tg(GFAP-tTA)110Pop/J (GFAP.tTA) mice derived from Jackson Labs (Sacramento, CA, USA; stock #005964) [24] with APP23 mice obtained from Novartis (Basel, Switzerland) [28], yielding double transgenic GFAP.tTA/APP23 mice with C57BL/6J background. To get triple-transgenic GFAP.tTA/(tetO)7.IKK2-CA/APP23 mice, GFAP.tTA/APP23 animals were combined with (tetO)7.IKK2-CA mice (NMRI background; with a luciferase reporter gene regulated by the same bidirectional promoter). The resulting triple-transgenic mouse model (called Ttg hereafter) exhibited a defined C57BL/6J:NMRI background (1:1) and allowed the expression of the human APP^SWE^ transgene in neurons under neuroinflammatory conditions induced by IKK2/NF-κB activation due to IKK2-CA transgene expression in astrocytes [29]. To keep the neuroinflammation suppressed during brain development, doxycycline (0.5 g/L; Sigma-Aldrich, Taufkirchen, Germany) was administered to all mice in drinking water containing 1% sucrose (Applichem, Darmstadt, Germany) until the age of four weeks. Expression of the IKK2-CA transgene was controlled by measuring luciferase activity in native protein extracts from the cerebellum, as described previously [30].

For the control group (Co), we used littermates, which had been genotyped as negative for all alleles, negative for APP23 and negative for either the GFAP.tTA or IKK2-CA transgene. APP23 animals were positive only for the APP23 transgene or positive for APP23 together with being positive for either the GFAP.tTA or IKK2-CA transgene. The GFAP/IKK2-CA group had to be positive for both the GFAP.tTA and the IKK2-CA transgenes, but negative for APP23. These mice were used as a control of astrocytic-driven inflammation alone, without the contribution of AD pathology, and were contrasted with the Ttg group. The Ttg mice bared all three transgenes together, in order to resemble inflammatory conditions in AD. Animals were kept in groups of 3–5 mice in a 12-h light–dark cycle and were given ad libitum access to food and water. All groups were used for all experiments, in order to evaluate and compare the effect of IKK2/NF-κB activation in astrocytes on the microglial response toward plaque degradation.

### 2.2. Protein Extraction

Tissue samples from different brain regions were snap-frozen in liquid nitrogen, pulverized in a mortar, aliquoted, and stored at −80 °C. For protein extraction, the brain powder was suspended in approximately three times its own volume in TNT buffer and mechanically homogenized. After 15 min incubation on ice, homogenization was repeated, and the lysates were centrifuged at 13,000 rpm at 4 °C for 25 min and stored at −80 °C. The supernatant which contains both soluble and membrane-associated proteins was transferred into a fresh tube and snap-frozen in liquid nitrogen. The remaining pellet containing cell debris and insoluble proteins was resuspended in approximately twice its own volume in 1× Lämmli-Urea (LU) buffer [31] and sonicated using a Diagenode (Liege, Belgium) Bioruptor^®^ sonication filled with iced water at high amplitude (3 cycles, with 30 s ON/30 s OFF). The lysates were centrifuged at 13,000 rpm at 4 °C for 10 min to remove the remaining debris, and the supernatant was transferred into a fresh tube, snap-frozen in liquid nitrogen, and subsequently stored at −80 °C.

### 2.3. Western Blot Analysis

For most applications with TNT extracts, equal amounts of protein (20–50 µg) were diluted with a quarter of the volume with LI-COR^®^ (Lincoln, NE, USA) protein-loading buffer according to the manufacturer’s instructions and then loaded on SDS-PAGE gels (12.5–15%) and transferred to 0.2 µm Amersham™ Hybond ECL (G&E Healthcare, Freiburg, Germany) nitrocellulose membranes. Afterward, membranes were blocked with 10 mL LI-COR^®^ casein-blocking buffer for 1 h at room temperature. The primary antibody (see below) in its corresponding dilution was added directly to the blocking buffer and incubated for 2–4 h at room temperature (RT) or overnight at 4 °C. After washing several times with TBS/ 0,1% Tween, the membranes were incubated with either an HRP-coupled secondary antibody from Santa Cruz^®^ (Dallas, TX, USA) or an IRDye coupled secondary antibody from LI-COR^®^ for 1 h at room temperature, both diluted in 0.5% Casein-blocking buffer in TBS.

Detection of IRDye-coupled blots was performed in an Odyssey infrared imaging system (LI-COR^®^), while the HRP-coupled blots were first incubated in WesternBright™ Quantum HRP substrate from Advansta (Menlo Park, CA, USA) for 5 min, and was performed either with classical X-ray films or a digital CCD-camera based system, “Intelligent Dark Box”, both from Fuji (Tokyo, Japan). Quantification was performed by LI-COR^®^ Image Studio™ Lite.

### 2.4. Histology and Immunostaining 

For histopathological analysis of Aβ deposits, the brains of the mice (12 months old) were processed as described previously [24,32], but without perfusion with paraformaldehyde (PFA). For Congo red staining, 20 µm thick brain sections were obtained using a microtome. Sections were processed exactly and quantified as published by Wilcock [33]. Images were taken with a Biorevo BZ-9000 from Keyence (Neu-Isenburg, Germany) with a DAPI, FITC, TexasRed^®^, Cy5^®^, and a bright field filter, 10–20× magnification, the BZ Viewer for capturing, and BZ Analyzer for merging. Images of stained sections were captured with equal exposure times per channel and the same graphical pre-processing within one experiment. For analysis and quantification, the ImageJ (Rasband, W.S., ImageJ, U. S. National Institutes of Health, Bethesda, Maryland, USA) and Image Pro-Plus (Media Cybernetics, Cambridge, UK) software were used.

PFA-fixed brain samples from the 1-year-old control and transgenic mice were obtained as previously reported [25]. Briefly, the animals were terminally anesthetized with ketamine and xylazine, transcardially perfused with a peristaltic pump (speed: 5 mL/min) by infusing 25 mL of ice-cold PBS followed by 50 mL of cold 4% PFA (pH 7.4). Brain samples were quickly dissected, post-fixed in 4% PFA for 18 h at 4 °C, washed in PBS, and dehydrated in 30% sucrose for 36 h. Samples were then snap-frozen in OCT (TissueTek), cryoprotected at −80 °C, and sectioned at −18 °C in a cryostat (Leica CM1950) at the thickness of 40 µm. Free-floating sections were subjected to heat-induced antigen retrieval with citrate buffer (pH 9). The sections were washed in PBS, blocked in PBS + 3% BSA + 5% donkey serum + 0.3% Triton-X, and incubated with the appropriate antibody combination from 48–72 h at 4 °C, followed by washing in PBS (45 min × 3) and incubation with a corresponding combination of fluorophore-coupled secondary antibodies for 2 h at room temperature; after washing, sections were mounted with Vectashield antifade mounting medium (Vector Laboratories, Burlingame, CA, USA). For each experimental group or timepoint, at least four animals were processed and analyzed.

### 2.5. Confocal Imaging and Image Analysis

**Figure 1:**
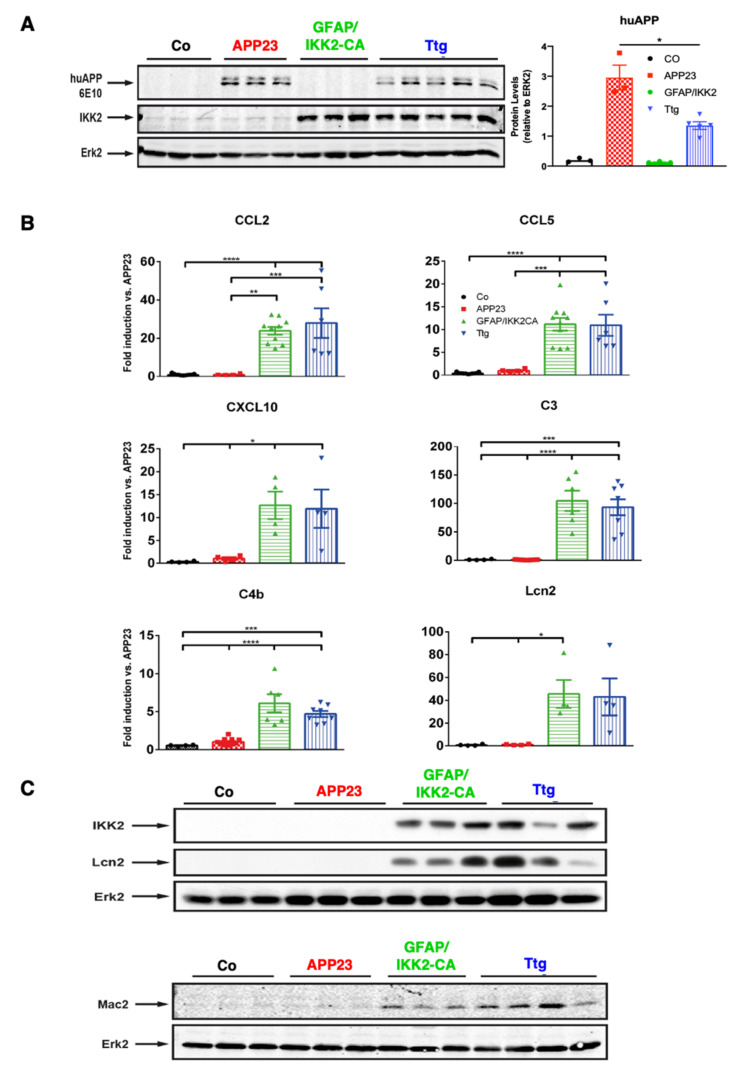
Functional characterization of the triple transgenic GFAP.tTA/tetO.IKK2-CA/APP23 (Ttg) mouse model of AD with chronic neuroinflammation as indicated by protein and gene expression. (**A**) Immunoblot of cortical TNT extracts from Co (Control), APP23, GFAP/IKK2-CA, and Ttg mice tested with antibodies against human (Tg)APP (6E10) and IKK1/2. Loading control is represented by Erk2. Quantification revealed that APP^SWE^ transgene expression is reduced by 50% in Ttg mice compared to APP23 mice. (**B**) qRT-PCR determination of cortical mRNA levels of pro-inflammatory chemokines and factors CCL2, CCL5, CXCL10, C3, C4b, and Lcn2 is shown. Expression is normalized to APP23 mice. Statistical analysis: ANOVA multiple comparison test (* *p* = 0.05; ** *p* = 0.01; *** *p* = 0.001; **** *p* = 0.0001). (**C**) Immunoblots of cortical TNT extracts from Co, APP23, GFAP/IKK2CA, and Ttg mice probed with antibodies against IKK1/2, Lcn2, and Mac2. Erk2 serves as a loading control. (APP = Amyloid precursor protein; IKK = IκB kinase complex; Lcn2 = Lipocalin 2; GFAP = Glial fibrillary acidic protein).

Confocal images were acquired using an LSM-710 (Carl Zeiss AG, Oberkochen, Germany) inverted microscope, fitted with a 20× air or 40× or 63× oil objective. Imaging parameters were set in order to obtain a signal for the immunostained antigen >150 while avoiding saturation in high-intensity structures. All images were acquired in correspondence with the gray matter of the cerebral cortex in the brain (Figure 1). Confocal stacks (5–10 optical sections) were collapsed in the maximum-intensity projection and the integrated mean gray value was obtained; fluorescence intensity was expressed in arbitrary units (a.u.) corresponding to the grayscale value (in 12-bits images, ranging from 0 to 4095). The quantification of the fraction of area occupied by GFAP+ and IBA1+ structures was performed as previously reported [25]; briefly, artifact-free ROI were considered in the superior and inferior cerebral cortex, and for each, we computed the ratio between the area displaying GFAP or IBA1 immunostaining above a set intensity threshold and the total area of the ROI. For quantitative analysis, a minimum of 4–8 artifact-free sections per mouse were analyzed.

### 2.6. Antibodies for Immunostaining and Immunoblotting

For immunoblotting, the following antibodies were used: rabbit anti-IKK1/2 (sc-7607), rabbit anti-Mac2/galectin-3 (sc-20157), rabbit anti-Erk2 (sc-154), HRP-conjugated goat anti-mouse (sc-2005), goat anti-rabbit (sc-2004), and donkey anti-goat (sc-2020) from Santa Cruz Biotechnology (Heidelberg, Germany). Mouse anti-β-amyloid (6E10, sig-39320) from Covance (Munich, Germany) and goat anti-Lcn2 (AF1857) from R&D (Minneapolis, MN, USA). For proper quantitative analysis, donkey anti-mouse and donkey anti-rabbit IRDye 680LT-conjugated secondary antibodies (#926-68022 and #926-68023) as well as donkey anti-mouse and anti-rabbit IRDye 800CW-conjugated secondary antibodies (#926-32212 and #926-32213) from LI-COR^®^ were used:

For immunofluorescent staining, the following antibodies were used: goat anti-GFAP and rabbit anti-Mac2/galectin-3 (sc-20157) from Santa Cruz Biotechnology (Heidelberg, Germany), chicken anti-GFAP (ab6476) from Abcam (Berlin, Germany), rabbit anti-Iba1 (019-19741) from WAKO (Neuss, Germany), and mouse anti-β-amyloid (4G8, sig-39320) from Covance (Munich, Germany). Alexa Fluor labeled secondary antibodies (488, 568/594, and 647) were obtained from Invitrogen (Waltham, MA USA) and DAPI was purchased from MERCK (Darmstadt, Germany).

### 2.7. RNA Extraction, cDNA Synthesis, and qRT-PCR

Cortical and hippocampal RNA were isolated using a PeqGOLD^®^ TriFast™ kit (peQlab, Darmstadt, Germany), for the genes without intron like (Tg)APP, RNA was first digested with RNase-free DNase (Qiagen, Hilden, Germany) and purified with the RNeasy^®^ Mini kit (Qiagen) according to the manufacturer’s instructions. For cDNA synthesis, the Transcriptor High fidelity cDNA synthesis kit (Roche, Penzberg, Germany) was used according to the manufacturer’s instructions but with only 0.5 µL instead of 1 µL reverse transcriptase mix and oligo-dT primers alone. To perform qRT-PCR, we used the LightCycler^®^ 480 Instrument (Roche) according to the manufacturer’s instructions with intron spanning primers (except for (Tg)APP) and mono-color hydrolysis probes designed by Roche’s Universal Probe Library UPL assay design center. As a reference gene, the housekeeping gene hypoxanthine-guanine phosphoribosyl transferase (Hprt) was used. Primer sequences and UPLs are available upon request.

### 2.8. Primary Cell Isolation and Cell Culture 

After the decapitation of neonatal mice (P3–P5), the brain was dissected, cerebellum, olfactory bulbs, and meninges were removed, and the brain was stored for a short time in ice-cold D-PBS (w/o Ca^2+^ and Mg^2+^) until use. The brains were washed three times in D-PBS (w/o Ca^2+^ and Mg^2+^), 1250 µL 10× Trypsin without EDTA per brain were added followed by 100 µL DNaseI. The samples were incubated for 5 min at RT and periodically inverted. Samples were washed three times in D-PBS (w/o Ca^2+^ and Mg^2+^), again 100 µL DNaseI was added, and the sample was homogenized with a Pasteur pipette. Four brains were merged, 20 mL DMEM/10% FBS/1% P/S was added, and the combined sample was centrifuged at 300× *g* at RT for 10 min. The pellet was resuspended in 10 mL DMEM/10% FBS/1% P/S, cells of two brains were seeded in a T75 flask or cells of one brain in a T25 flask. Cells were incubated in a humidified incubator at 37 °C with 5% CO_2_ for the entire time and treated with EtOH/4-OHT (100 nM) on days 10, 12, and 14. Cells were harvested on day 17. For harvesting, flasks were shaken for 2 min by hand to gently detach the microglia cells from the astrocytic monolayer. The supernatant containing microglia cells was centrifuged for 5 min at 300× *g* and 4 °C. The cell pellet was either further processed for flow cytometry analysis or snap-frozen in liquid nitrogen (N2) and stored at −80 °C until further analysis.

Primary astrocytes and microglia from adult mice were isolated using the Adult Brain Dissociation Kit, OctoMACS^®^ with heaters, ACSA-2 and Cd11b beads as well as MS columns in a magnetic field (Miltenyi, Germany) according to the manufacturer’s protocol. In brief, brains were dissected, enzymatically digested, and debris was removed. The obtained cell suspension was labeled with ACSA-2 beads, washed, and then added to an MS column. The pellet of purified ACSA-2 positive astrocytes was snap-frozen in liquid nitrogen (N2) and stored at −80 °C until further analysis. The flowthrough and three washing volumes of the column containing unlabeled cells were combined and labeled again, this time with CD11b beads, washed and purified using an MS column. The pellet of purified CD11b positive microglia was snap-frozen in liquid nitrogen (N2) and stored at −80 °C until further analysis.

### 2.9. Statistical Analysis

Statistical analysis was performed with Prism software (GraphPad, La Jolla, CA, USA). One-way ANOVA followed by the Tukey’s multiple comparison test or Student’s *t*-test were used as indicated in the figure legends, all data are shown as mean ± SEM.

## 3. Results

### 3.1. Establishment of a Novel APP23 Mouse Model of AD (Ttg) Combined with Conditional Activation of Neuroinflammation 

We combined our GFAP.tTA/tetO.IKK2-CA [25,29,34] mouse model with the well-characterized APP23 model of AD to investigate how IKK/NF-κB-driven neuroinflammation affects the pathogenesis of AD. APP23 mice express human APP harboring the Swedish double mutation KM670/671NL (APP^SWE^) under the control of the Thy1.2 promotor [28]. In the APP23 model, first amyloid deposits were observed at the age of six months, followed by the upregulation of complement system factors and gliosis. The generation of the GFAP.tTA/tetO.IKK2-CA/APP23 triple-transgenic mouse model (Ttg) allows the expression of the APP^SWE^ transgene in the context of chronic postnatal neuroinflammation.

We turned off IKK2-CA transgene expression during development until the age of four weeks by doxycycline application and, thereafter, the transgene was activated by doxycycline withdrawal. In this experimental paradigm, GFAP.tTA/tetO.IKK2-CA mice develop prominent neuroinflammation throughout the central nervous system (CNS), starting at the age of eight weeks. We first evaluated whether the combination of the transgenes affects hAPP expression. To this end, quantitative RT-PCR analysis revealed that APP^SWE^ transgene transcription is comparable between single transgenic APP23 mice and the combined Ttg model (Figure S1A). Subsequent Western blot analysis using cortical protein extracts confirmed that APP^SWE^ transgene expression did not affect transgenic IKK2-CA expression (Figure 1A).

The functional consequences of IKK2-CA transgene expression and successive NF-κB activation in astrocytes were demonstrated by the significant upregulation of inflammatory marker genes, such as the chemokines CCL2, CCL5, and CXCL10, and the complement factors C3 and C4b, which was confirmed in both the cortex (Figure 1B) and the hippocampal area (Figure S1B). We detected a prominent pro-inflammatory gene expression profile in GFAP/IKK2-CA and Ttg mice, the groups with IKK2-CA transgene expression, compared to the control and APP23 animals, confirming an aggravated neuroinflammatory milieu in these groups. Furthermore, lipocalin 2 (Lcn2), a marker for ongoing neuroinflammation [35], is similarly upregulated in Ttg and GFAP/IKK2-CA mice on both mRNA (Figure 1B and Figure S1B) and the protein level (Figure 1C). Associated with the elevated pro-inflammatory gene expression, we also identified an increase of Galectin-3/Mac-2, a marker of macrophage activation associated with a phagocytic phenotype of microglial cells [36,37] and microgliosis, by Western blot analysis and immunofluorescence staining (IF) in both GFAP/IKK2-CA and Ttg mice (Figure 1C and Figure S1C).

**Figure 2:**
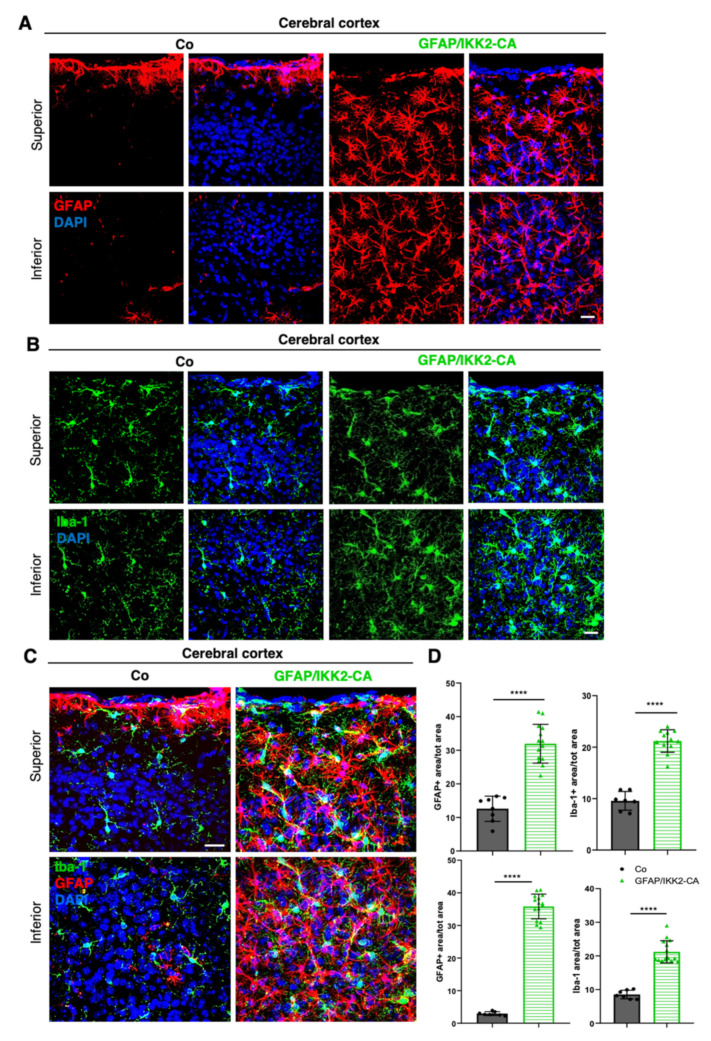
Selective activation of IKK2/NF-κB signaling in astrocytes induces strong astrogliosis and microgliosis. Representative pictures showing IF staining of astrocytic marker GFAP (**A**) and microglial cells marker IBA-1 (**B**) in the superior and inferior cerebral cortex of control mice and GFAP/IKK-CA littermates. (**C**) High-magnified pictures depicting co-IF staining of GFAP and IBA-1 markers in the superior cerebral cortex. The panel displays interaction between the two types of glial cells and underlines the increase in number and area covered by astrocytic and microglial cells in GFAP/IKK2-CA mice in relation to Co littermates. Nuclei are labeled with DAPI (blue). (**D**) Quantitative analyses show a significant increase in GFAP+ astroglia (**A**) and IBA-1+ microglia area (**B**) in the GFAP/IKK2-CA mice compared to control mice. Cryo-sections thickness = 40 µm; Scale bar = 20 µm. Statistical analysis: ANOVA multiple comparison test (**** *p* = 0.0001).

To assess astrogliosis and microgliosis induced by astrocytic IKK2/NF-κB activation, we performed immunofluorescent (IF) analyses using GFAP/IBA-1 co-staining. Consistent with our former characterization of gliosis in the spinal cord of ALS mice [25], we identified prominent astrogliosis and microglia expansion in the cerebral cortex of GFAP/IKK2-CA mice (Figure 2A–D) initiated by the activation of IKK2/NF-κB signaling in astrocytes. This was further confirmed for the Ttg situation when the cortical area (Figure S2A,B) and the hippocampus (Figure S2C) were analyzed in comparison to APP23 mice.

### 3.2. Astrocyte-Driven Neuroinflammation in APP23 Mice Reduces Plaque Number and Ameliorates Amyloid Plaque Burden

We then investigated the consequences of astrocytic IKK2/NF-κB activation on the Aβ plaque formation in one-year-old APP23 and Ttg animals. For this purpose, we visualized Aβ plaques by staining the whole brain with Congo red, which reacts with compacted amyloid proteins with a β-sheet secondary structure, such as Aβ and α-synuclein [38]. This allowed the quantification of plaque number, area, and size. Remarkably, we detected a more than ten-fold reduction in plaque number and an almost twenty-fold decrease in the total area occupied by the plaques. This effect was accompanied by a slight decrease in individual plaque size when comparing APP23 with Ttg mice (Figure S3A).

**Figure 3:**
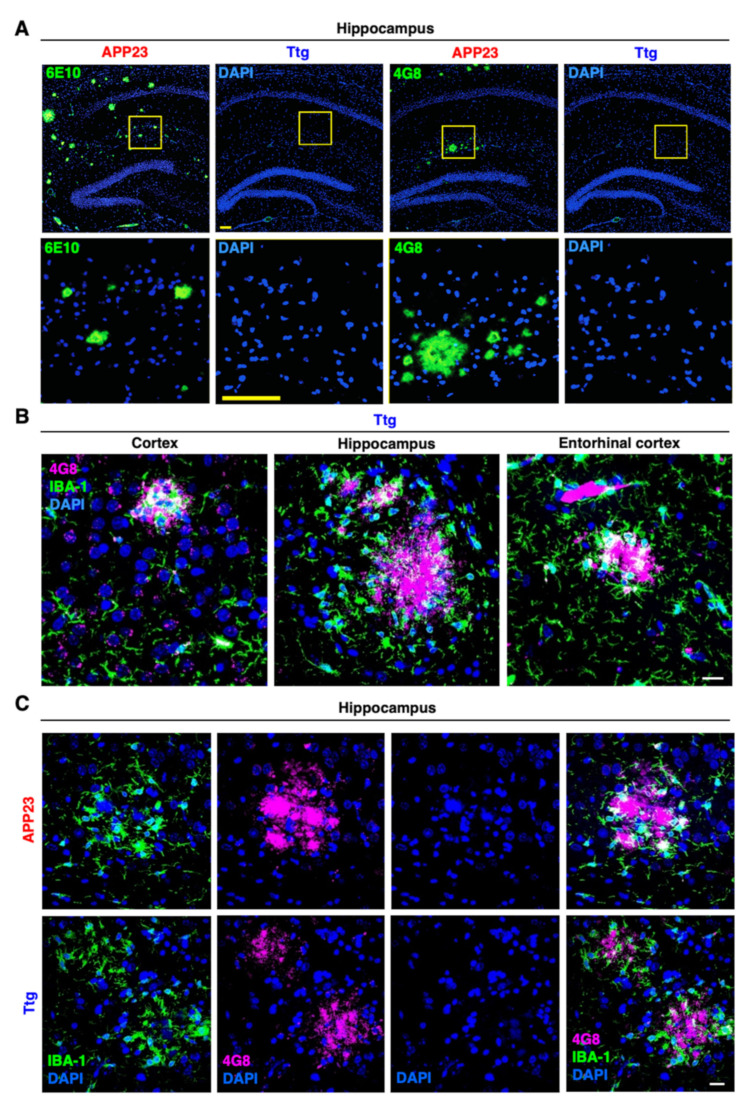
Plaque number and amyloid burden are reduced in Ttg mice compared to APP23 model without IKK2/NF-κB signaling in astrocytes. (**A**) Representative panel displaying decreased immunoreactivity to Aβ 6E10 (green, left) and 4G8 antibody (green, right) in the hippocampal area of Ttg mice compared to APP23 littermates at 12 months of age. (**B**) IF staining of Aβ 4G8-positive plaques (magenta) in relation to IBA-1-positive microglial cells (green), localized in different brain regions, namely Cortex, Hippocampus, and Entorhinal cortex of the Ttg animals. (**C**) Panel displaying different morphology and structure of Aβ 4G8-positive plaques (magenta), which appear smaller and less intensively stained in the Ttg mice compared to the APP23 littermates. Plaques are surrounded by IBA-1-positive microglia (green), which seem less ramified in Ttg mice compared to APP23 littermates. Nuclei are labeled with DAPI (blue). Pictures are from paraffin section (7 µm thickness). Scale bar = 20 µm.

**Figure 4:**
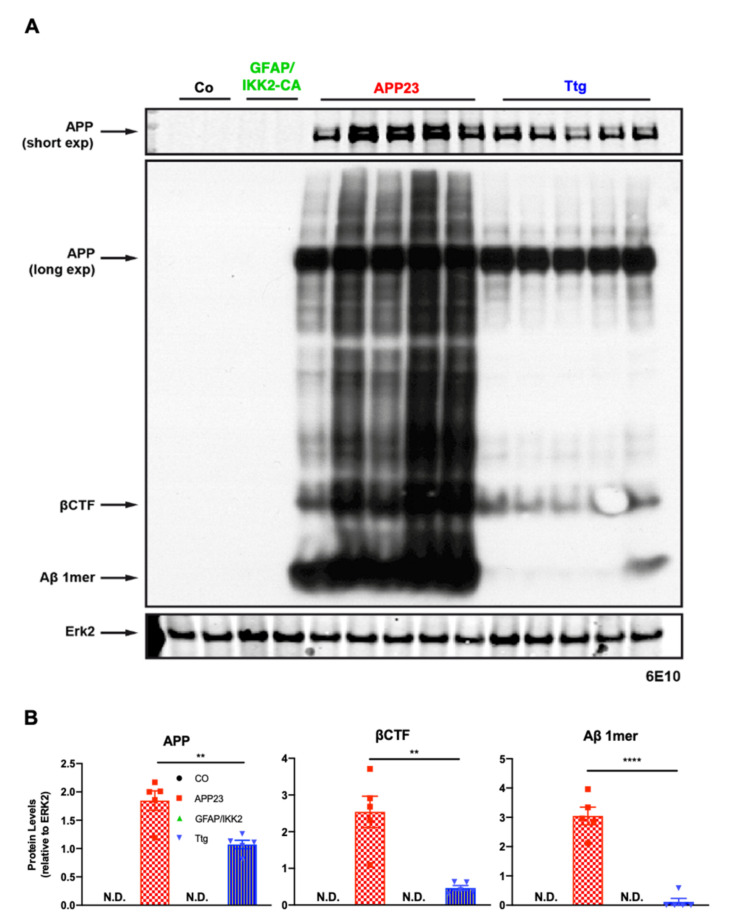
Astrocytic-driven neuroinflammation in the APP23 animal model induces amyloid protein level reduction in Ttg mice. (**A**) Western blot assessment of insoluble Lämmli-Urea (LU) protein fraction of Aβ burden in cortical extracts from Co, APP23, GFAP/IKK2CA, and Ttg mice, tested with antibody against human APP (6E10). Long exposure immunoblot shows expression of huAPP (6E10), β-CTF and monomeric Aβ in a “smear” form. (**B**) Quantification of huAPP (short exposure) revealed that APP levels are decreased by 50% in Ttg mice compared to APP23 littermates, whereas β-CTF and monomeric Aβ levels were massively decreased. Quantification of huAPP (6E10), β-CTF and monomeric Aβ was normalized to Erk2 (used as loading control). Statistical analysis: Student’s *t*-test (** *p* = 0.01; **** *p* = 0.0001); Exp = exposure; N.D. = Not detectable; βCTF = Beta C-terminal fragment; Aβ 1mer = monomeric Aβ.

To detect APP-derived plaques, and to prove the nature and composition of the Congo red-stained amyloid deposits, we next performed IF staining with the Aβ-specific 6E10 (Figure 3A) and 4G8 (Figure 3B,C) antibodies. IF staining also confirmed the prominent reduction in plaque number in the hippocampal area (Figure 3C). Moreover, we showed that the plaque morphology in the Ttg mice seemed more compact and circumscribed than the diffuse-appearing plaques found in the APP23 model when observed after Congo red staining under bright field and polarized light conditions (Figure S3B).

Additionally, we demonstrated that the differences in plaque morphology coincided with changes in local gliosis surrounding the plaques. We showed a strong and more homogenous brain-wide activation of microglia and astrocytes in GFAP/IKK2-CA and Ttg animals compared to the AD-specific pattern of astrogliosis and microgliosis observed in APP23 mice. Indeed, APP23 mice were characterized by numerous GFAP- and IBA-1-positive glial cells encircling the plaques (Figure S4A–E).

To confirm the reduction in the amyloid load in Ttg mice, and to determine the presence of APP cleavage products, we extracted, in addition to the soluble TNT (Figure 1A), an insoluble Lämmli-Urea (LU) protein fraction from brain tissue. Immunoblot analyses of extracts containing insoluble Aβ revealed a sharp reduction in monomeric Aβ together with different multimeric forms (“smear”; Figure 4A), in line with the reduced plaque number observed by Congo red staining (Figure S3A). In addition, we detected lower levels of β-CTF (C-99), an intermediate fragment of APP processing and direct precursor of Aβ (Figure 4A). However, Ttg mice showed a reduction of approximately 50% in the overall amount of full-length APP (short exposure) in the insoluble fraction compared to APP23 littermates (Figure 4A).

### 3.3. Expression of Enzymes Involved in APP Processing and Proteolytic Aβ Degradation Is Not Prominently Altered by Astrocytic IKK2/NF-κB Activation

**Figure 5:**
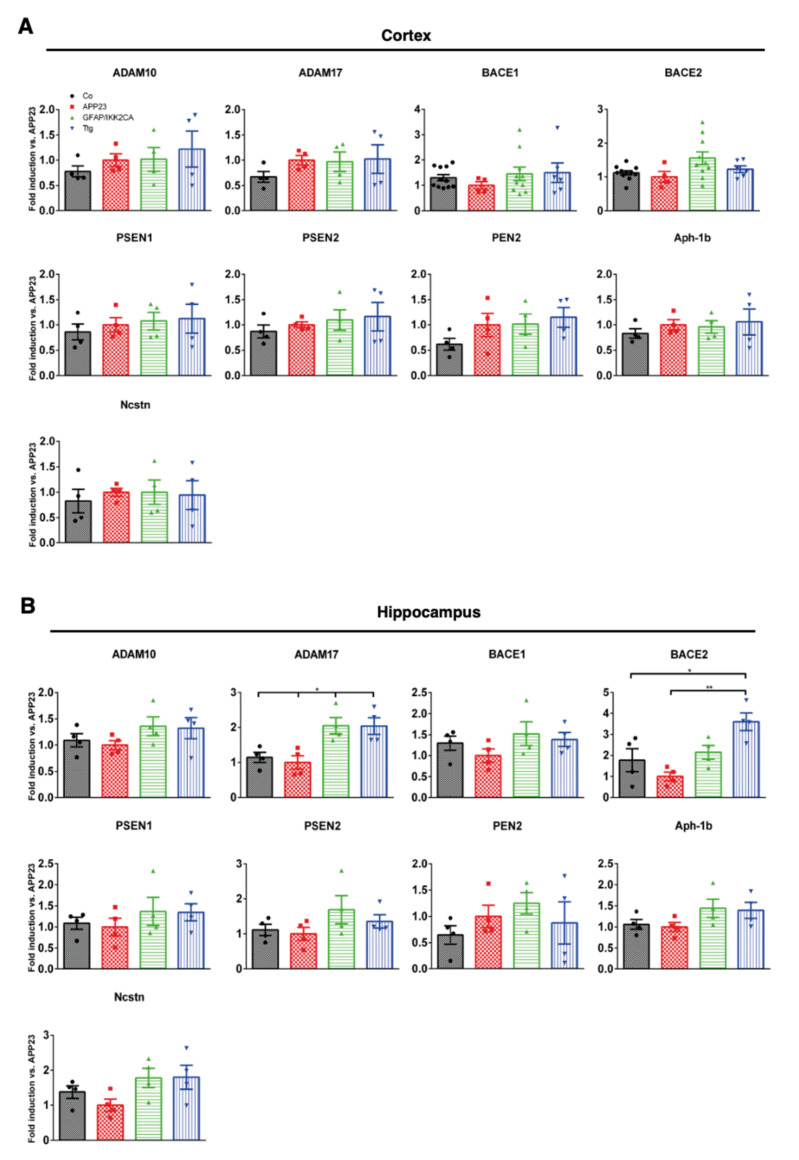
Gene expression of enzymes involved in the processing of APP is selectively affected in the hippocampus of Ttg mice. qRT-PCR analysis of ADAM10, ADAM17, BACE1, BACE2, PSEN1, PSEN2, PEN2, Aph1b, and Ncstn mRNA levels in the cortex (**A**) and hippocampus (**B**) of Co, APP23, GFAP/IKK2-CA, and Ttg mice. In contrast to the cortical region, quantitative analysis shows a significant increase in ADAM17 and BACE2 gene expression in the hippocampal region of GFAP/IKK2CA and Ttg mice compared to Co and APP23 littermates. Expression is normalized to APP23. Statistical analysis: ANOVA multiple comparison test (* *p* = 0.05; ** *p* = 0.01). ADAM10 = ADAM Metallopeptidase Domain 10; ADAM17 = ADAM Metallopeptidase Domain 17; BACE1 = Beta-Secretase 1; BACE2 = Beta-Secretase 2; PSEN1 = Presenilin 1; PSEN2 = Presenilin 2; PEN2 = Gamma-Secretase Subunit PEN-2; APH1B = aph-1 homolog B, gamma-secretase subunit; Ncstn = Nicastrin.

To examine whether the reduction in the overall Aβ plaque load in Ttg compared to APP23 animals resulted from less Aβ generation or an enhanced Aβ turnover, we considered the proteolytic processing of the amyloid precursor protein (APP) and Aβ biogenesis [23]. We wondered whether the amyloidogenic pathway remained intact or whether it is shifted toward the non-amyloidogenic pathway in the context of astrocyte-initiated neuroinflammation. For this purpose, we quantified the expression levels of the α-secretase isoforms ADAM10 and ADAM17, β-secretase isoforms BACE1 and BACE2, and γ-secretase subunits PSEN1, PSEN2, PEN2, Aph-1b, and Ncstn [39] via qRT-PCR (Figure 5A,B).

In the cortex (Figure 5A), no significant differences could be detected in the gene expression levels of components of the APP processing machinery (ADAM10, ADAM17, BACE1, BACE2, PSEN1, PSEN2, PEN2, Aph-1b, and Ncstn) between the different groups of mice, suggesting that the proteolytic processing of APP is not altered in Ttg and APP23 mice (Figure 5A). However, in the hippocampal region, we found a two–threefold increase of ADAM17 and BACE2 gene expression (Figure 5B).

We also investigated the expression of metalloproteinases like neprilysin and the insulin-degrading enzyme (Ide) [40], which are involved in the cleavage of monomeric Aβ, and matrix-metalloproteinases MMP2 and MMP9, engaged in the degradation of Aβ fibrils [41], in the cortex and hippocampus. Precisely, in the cortex, we detected no significant changes in the mRNA levels (Figure S5A,B), whereas a 50% reduction of IDE and three-fold increase of MMP2 was measured in the hippocampus of Ttg mice compared to APP23 animals (Figure S5B).

### 3.4. Astrocytic NF-κB Activation Promotes Microglia Polarization toward a Phagocytic-Proficient Phenotype 

**Figure 6:**
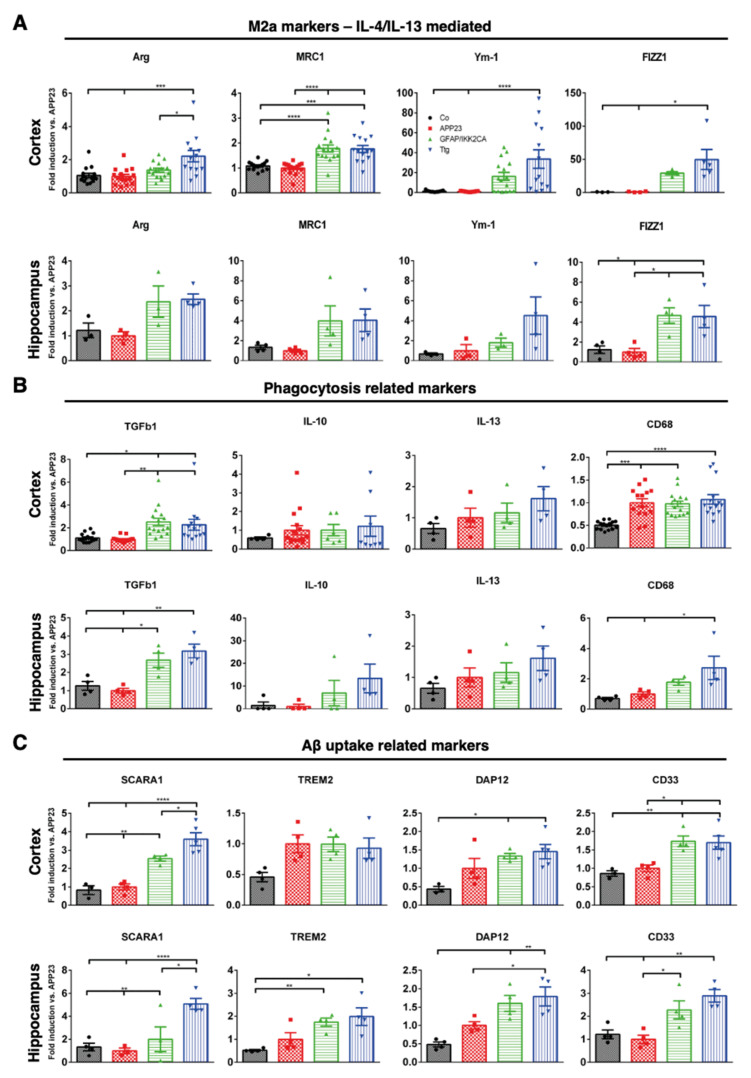
Astrocytic NF-κB activation initiates a neuroinflammatory response exhibiting an M2-like polarization status. (**A**) qRT-PCR analysis of M2a markers (IL-4/IL13) mediated, (**B**) qRT-PCR analysis of markers indicative for M2-type phagocytic polarization and (**C**) qRT-PCR analysis of genes involved in Aβ binding, uptake, and clearing in cortical and hippocampal samples derived from Co, APP23, GFAP/IKK2-CA, and Ttg mice, respectively as indicated. Results show strong upregulation of Arg, MRC1, Ym-1, FIZZ1, and TGFb1 in both cortical and hippocampal samples of Ttg mice, indicative of an M2-like polarization of microglia. Ttg mice also display an increased level of CD68 expression, a marker for phagocytic microglia together with elevation of factors involved in the Aβ binding and clearance. Expression is normalized to APP23. Statistical analysis: ANOVA multiple comparison test (* *p* = 0.05; ** *p* = 0.01; *** *p* = 0.001; **** *p* = 0.0001). Arg = arginase I; MRC1 = mannose receptor, C type 1; Ym1 = chitinase-like 3; FIZZ1 = found in inflammatory zone; TGFb1 = transforming growth factor β1; IL = interleukin; CD68 = cluster of differentiation 68; SCARA1 = scavenger receptor class A member 1; TREM2 = Triggering receptor expressed on myeloid cells 2; DAP12 = DNAX activation protein of 12 kDa; CD33 = Siglec-3 = sialic acid-binding immunoglobulin-like lectin.

Ttg mice express a normal APP processing machinery in the cortex and a two–threefold increase of ADAM17 and BACE2 gene expression in the hippocampus, but overall, they exhibit a pronounced activation of microglia (Figure S2A–C). We, therefore, analyzed whether these cells acquire a specific functional state. For this, we determined the gene expression level of established markers indicative of either the pro-inflammatory (Figure S6A) or M1-like (IL-1β, IL-6, and iNOS) or the anti-inflammatory or M2-like phenotype (Arg-1, MRC1, Ym-1, and FIZZ1) [42] in cortical and hippocampal brain samples using qRT-PCR. While M1-type markers were mildly but significantly induced (Figure 6A), we could detect a very prominent upregulation of M2-type genes in the whole cortex, reaching up to 40- and 50-fold induction for Ym-1 and FIZZ1 compared to APP23 mice. This effect is less pronounced in the hippocampus, where only Fizz1 expression was significantly upregulated in Ttg mice compared to their APP23 counterparts (Figure 6A).

**Figure 7:**
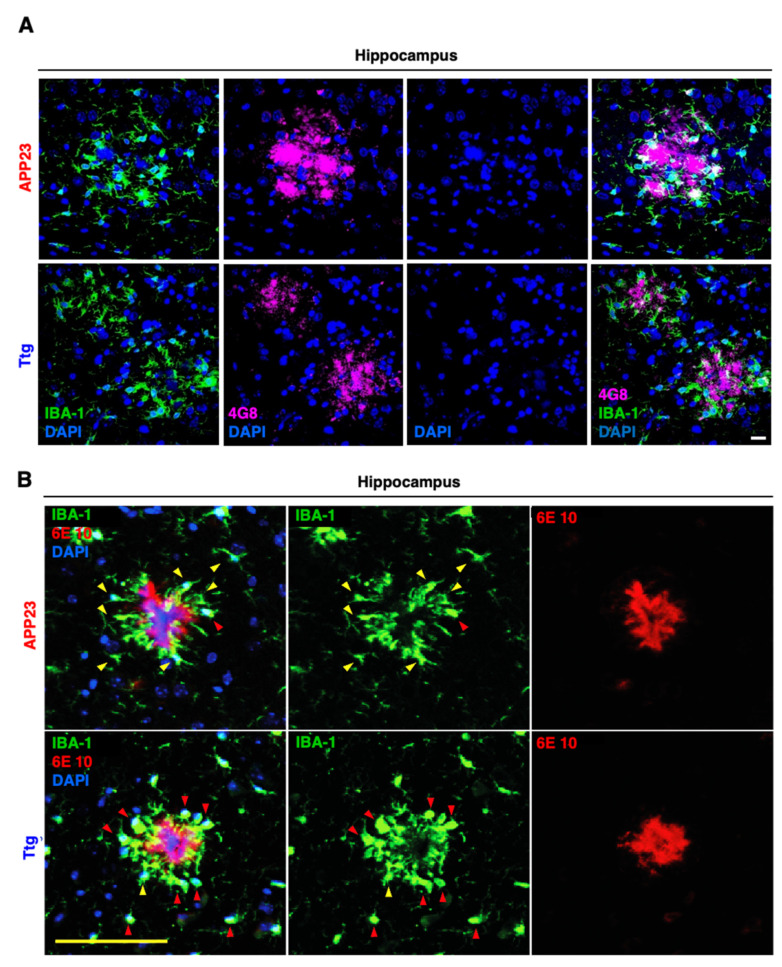
Ttg mice display a phagocytic-proficient Iba1-positive microglia morphology surrounding Aβ plaques. (**A**) Representative panel depicting IF staining of microglial cells (IBA-1 in green) surrounding Aβ plaques (in magenta). Plaques stained with the 4G8 antibody appear more dissolved and smaller in the Ttg mice compared to the APP23 littermates. Scale bar = 20 µm. (**B**) Representative panel depicting different microglia morphology in Ttg mice compared to APP23 mice. Microglial cells (IBA-1 in green) surrounding the plaques (6E10 in red) appear more rounded and amoeboid, with a compact structure (red arrowheads) in Ttg animals compared to APP23 littermates, suggesting enhanced phagocytic activity. In the APP23 group, the yellow arrows indicate a filamentous microglia shape with a less compact structure. Nuclei are labeled with DAPI (blue). Scale bar = 100 µm.

Several factors, e.g., TGF-β, IL-13, and IL-10 have been described as inducing an M2 microglia phenotype [43,44,45]. We observed a two-fold induction of TGF-β expression in the cortex and an even higher expression in the hippocampus of Ttg and GFAP/IKK2CA mice, while IL-13 and IL-10 remained unchanged (Figure 6B). In the hippocampus, we also determined a three-fold induction of CD68, a marker for activated, phagocytic microglia (Figure 6B). Importantly, we found an upregulation of factors responsible for improved microglia-mediated Aβ-clearance, such as SCARA1 (CD204), TREM2, DAP12, and CD33 [46] in both the cortex and hippocampus of Ttg mice (Figure 6C). SCARA1, a phagocytic microglial receptor that is known to mediate the uptake of Aβ [47], was upregulated almost four-fold in the cortex and five-fold in the hippocampus. TREM2, which is essential for the microglial response to the plaques [17,48], was only increased in the hippocampus, while its adaptor protein DAP12, involved in promoting phagocytosis, was upregulated in both the cortex and hippocampus (Figure 6C). In contrast, CD33, a transmembrane, phagocytosis-regulating protein with controversial roles in Aβ uptake [49,50], was only slightly upregulated in the cortex, but three-fold upregulated in the hippocampus (Figure 6C). Overall, the determining gene expression pattern indicates a clear enrichment of receptors promoting phagocytosis. Moreover, we observed signs of morphological changes in plaque-associated microglia. Microglial cells surrounding the plaques in the Ttg model displayed thicker processes and a more compact appearance compared to the more filamentous microglia in the APP23 animals (Figure 7A,B). This correlates more with a phagocytic-proficient phenotype rather than an M1- or resting state-type [51].

**Figure 8:**
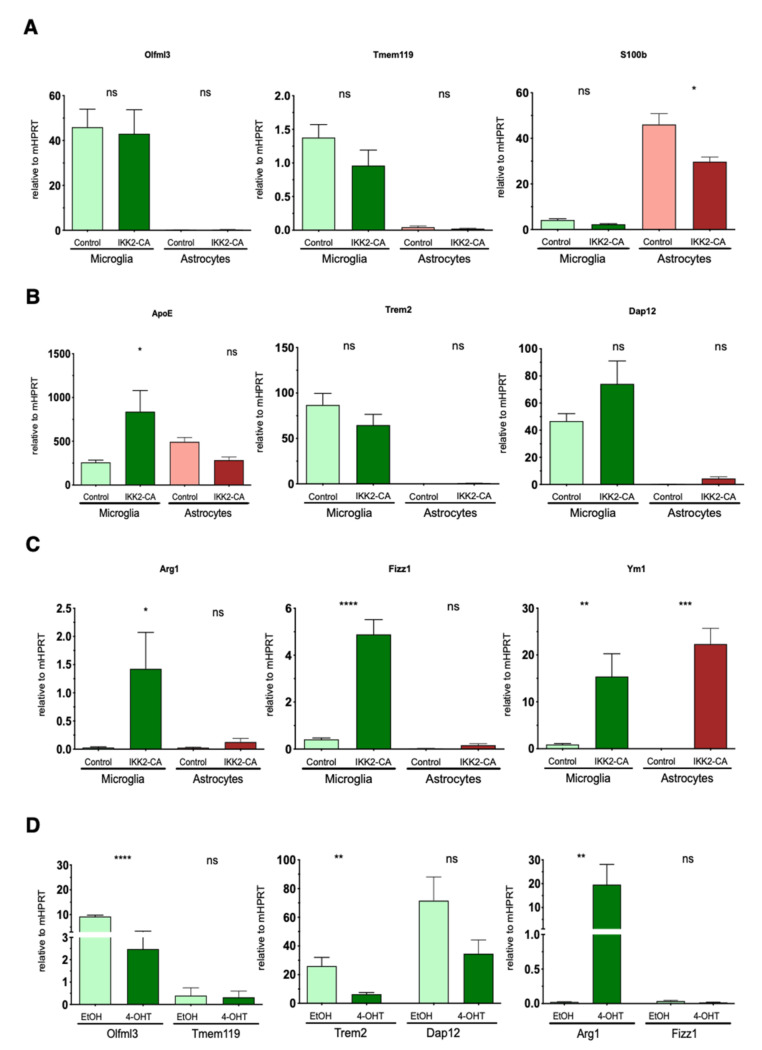
Primary microglial cells isolated from IKK2-CA^GFAP^ mice express an elevated M2-like gene profile. (**A**) qRT-PCR gene expression analysis of cell-specific identity markers for microglia (Olfm3 and Tmem119) and astrocytes (S100β confirmed cell-type-specific purification of primary cells derived from Control and GFAP/IKK2-CA animals. (**B**) ApoE, Trem2, and Dap12 qRT-PCR gene expression analysis of primary isolated microglial and astrocytic cells derived from Co and GFAP/IKK2-CA mice. (**C**) qRT-PCR gene expression analysis of M2-like markers Arg1, Fizz1, and Ym1 expressed in primary microglial and astrocytic cells derived from Co and GFAP/IKK2-CA littermates. (**D**) qRT-PCR analysis was performed with microglia cells (mixed glia culture) derived from the CX3CR1-CreERT2/CAG-LSL-IKK2-CA model to determine expression of Olfm3, Tmem119, Trem2, Dap12, and M2-like markers like Arg1, Fizz1 in control cells (ETOH) compared to microglia cells with activated IKK2/NF-κB signaling (4-OHT). (**B**,**C**) Trem2 and Dap12 were found upregulated in CD11b+ microglia cells and were almost not detectable in astrocytes. Arg1, Fizz1, and to a lower extent ApoE, were strongly expressed only in IKK2-CA^GFAP^-derived microglia cells, whereas Ym1 was prominently upregulated in both microglia and astrocytes of IKK2-CA^GFAP^ mice. (**D**) Upon activated IKK2/NF-κB signaling (4-OHT), levels of the microglia marker genes Olfm3 and Tmem119 together with Trem2 and Dap12 become reduced. M2-like markers like Fizz1 (and Ym1) were expressed at the detection limit whereas Arg1 was upregulated upon IKK2/NF-κ activation. Expression is normalized to the HPRT housekeeping gene. Statistical analysis: ANOVA multiple comparison test (* *p* = 0.05; ** *p* = 0.01; *** *p* = 0.001; **** *p* = 0.0001; ns = non-significant). HPRT = hypoxanthine phosphoribosyltransferase.

To assess further the hypothesis of a specific functional microglia state induced by IKK2/NF-κB activation in astrocytes, we determined the cell-type-specific gene expression pattern in our model system. For this purpose, we first isolated primary astrocytes expressing astrocyte cell surface antigen-2 (ACSA-2) from total brain tissue derived from one-year-old IKK2-CA^GFAP^ mice. Subsequently, we purified CD11b+ cells (representing mainly microglia) from the flow-through and performed a side-by-side gene expression analysis of both cell populations. Here, the CD11b+ cells derived from IKK2-CA^GFAP^ mice revealed prominent changes in anti-inflammatory marker genes resembling an M2-like polarization status with upregulation of Arg1, Fizz1, and Ym1 together with ApoE (Figure 8A–C; Table 1), compared to CD11b+ cells derived from the control littermates.

Finally, we asked whether a potential astrocyte–microglia crosstalk signaling culminates in the immediate activation of IKK/NF-κB signaling in microglial cells. For this, we performed ex vivo mixed glial cell culture experiments to test whether direct IKK2/NF-κB activation in microglia is able to mimic the polarization program initiated by astrocytic NF-κB using a novel transgenic mouse model based on the Cre/LoxP-system. In detail, we isolated primary glial cells from the forebrain of CX3CR1-CreERT2/CAG-LSL-IKK2-CA mice (P3-P5), which allow tamoxifen-mediated Cre-recombinase activation, finally leading to the expression of a constitutively-active IKK2-CA allele in microglia cells. Subsequent investigation of primary microglia (derived from the mixed glia culture) with transgenic IKK2-CA-mediated NF-κB activation revealed a gene expression pattern resembling a pro-inflammatory phenotype (Figure 8D, Table 1). This is depicted by the strong upregulation of the pro-inflammatory genes C3, Ccl2, Ccl5, Il1b, and Lcn2 in this type of microglia cell, derived from the CX3CR1-CreERT2/CAG-LSL-IKK2-CA model. In contrast, these genes are only mildly modulated in microglia derived from IKK2-CA^GFAP^ mice with activated IKK2/NF-κB signaling in astrocytes. With the exception of Arg1, which is strongly upregulated, other M2-like marker genes, such as Mrc1 (CD206), were downregulated or not expressed, such as Fizz1 and Ym1, in microglia cells with direct IKK2/NF-κB activation (Figure 8D, Table 1). In summary, these data indicate that direct IKK2/NF-κB activation in primary microglia cells is sufficient to induce a pro-inflammatory differentiation program.

## 4. Discussion

**Figure 9:**
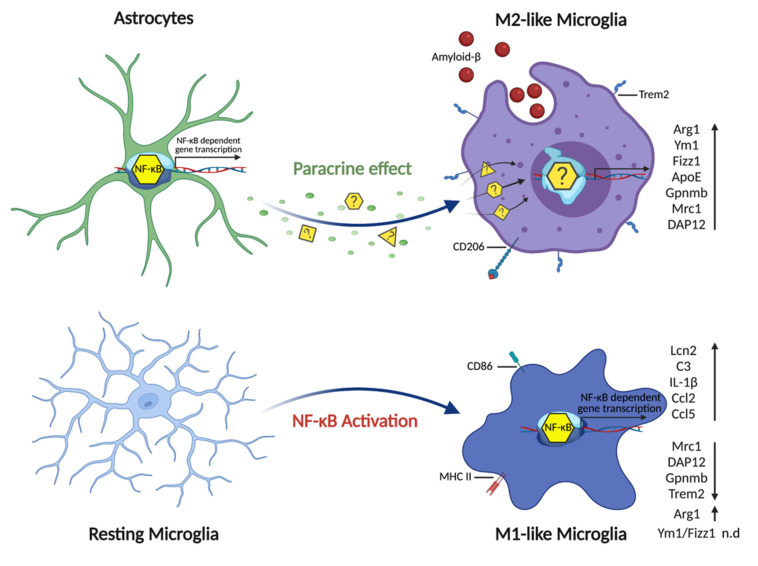
Activation of IKK2/NF-κB signaling in astrocytes promotes M2-like microglia polarization. IKK2/NF-κB activation restricted to astrocytes induces a genetic program that may promote the release of paracrine acting factors or create a condition, which enhances microglial differentiation toward a phagocytic phenotype as indicated by upregulation of typical M2-like marker gene expression. As an outcome, β-amyloid plaque deposition is significantly reduced in the context of the APP23 mouse model of AD. On the cellular level, microglial cells change their morphology from a resting ramified status to a globose-amoeboid one, reflecting the shape known to be competent to phagocyte amyloid deposits within the brain. On the contrary, cell-intrinsic NF-κB activation in primary microglial cells induces a pro-inflammatory phenotype with massive upregulation of inflammatory factors together with the downregulation of specific phagocytic markers as indicated.

In the present study, we could demonstrate that a neuroinflammatory response driven by astrocytic IKK2/NF-κB activation is able to significantly reduce the number of Aβ plaques and the overall Aβ burden in the cortex and hippocampus of one-year-old APP23 mice. Our evidence suggests that this phenotype is not due to major changes in APP transgene expression and/or in the Aβ processing machinery. Rather, we identified a specific microglia polarization state, which is characterized by an increased expression of marker genes associated with a phagocytosis-proficient phenotype, suggesting that an increased plaque clearance by these cells might be the critical mechanism accounting for the reduction in amyloid β plaque deposition (Figure 9).

Recent single-cell RNA-seq studies provided clear evidence for diverse subpopulations and activation states of microglial cells which goes far beyond the classical M1–M2 paradigm of functional protective (M2) and detrimental (M1) microglia states. In addition, multiple gene expression profiles have been identified and associated with specific functions in the neurodegeneration process in the course of Alzheimer’s disease. However, the exact pathways and mechanisms regulating the differentiation process of microglia from one functional state to another are less understood but it is of high interest for potential therapeutical approaches. Our findings suggest that IKK2/NF-κB-mediated astrocyte activation induces a genetic program leading to a reactive astrocyte state that may promote the release of paracrine acting factors, which act on the microglial polarization state. So far, the nature of this potentially paracrine-acting factor is not clarified. We cannot exclude the contribution of cell death of astroglia, which as a consequence converts microglia to a novel phagocytic phenotype, although we have not observed obvious signs of astrocytic cell death. Notably, in our previous study [25], using GFAP/IKK2-CA mice in the context of ALS, we proposed WNT family members as paracrine effectors to be involved in the expansion of microglia in the spinal cord, and the administration of the Porcupine WNT acyltransferase inhibitor C59 indeed decreased microglia density. Independent of this point, our findings underline the importance of astrocytic-driven neuroinflammation in AD pathogenesis [1,52].

Reactive gliosis is a hallmark of AD histopathology which is reflected by the morphological changes of glial cells as well as the proliferation of microglia cells and astrocytes that additionally exhibit hypertrophic processes and elevated GFAP expression. Overall, microgliosis and astrogliosis are common features of AD pathogenesis; however, it is not clear which histopathological and underlying molecular changes represent a beneficial, detrimental, or bystander state of glial cells in the neurodegenerative process. Interestingly, a large number of identified risk genes for AD were found to be highly expressed and several are also selectively expressed by microglia suggesting that specific functions of these cells are able to protect against the incidence of AD [4,5,53]. In addition to apolipoprotein E (ApoE, risk allele ε4) [54,55], these include the triggering receptor expressed on myeloid cell 2 (TREM2), complement receptor 1 (CR1), ATP-binding cassette sub-family A membrane 7 (ABCA7), CD33, and various other risk genes [53,56,57,58]. These findings support the idea that microglia can acquire a specific functional state depending on the adequate expression and function of AD risk genes. Consistent with this notion, the TREM2 risk mutation R62H, for example, is a loss-of-function mutation that impairs healthy microglia activation and phagocytosis [59,60,61,62,63] and, therefore, the combination of APP transgenic mice with Trem2 inactivation leads to an increased plaque burden with aging [64]. In our model, we found high expression of Trem2 in isolated microglia cells derived from both the control and the mice with astrocytic IKK2/NF-κB activation suggesting normal Trem2-mediated microglial activation and phagocytosis in our model.

Moreover, detrimental effects of microglia activation have been identified, in particular, microglia can mediate aberrant synaptic pruning via complement-dependent mechanisms finally leading to synapse loss and cognitive decline [5,53,65]. We found the complement factors C1qα and C1qβ to be strongly expressed in the isolated CD11b+ microglia population but without any difference between the control and microglia derived from mice with astrocytic IKK2/NF-κB activation. In contrast, we observed a selective upregulation of the complement factor C3 in both purified astrocytes with activated IKK2/NF-κB as well as in the IKK2-CA^GFAP^ microglia compared to the controls. Thus, C3-driven microglia-dependent synapse engulfment is a plausible outcome and needs to be addressed in future studies in order to clarify whether the improved Aβ clearance capabilities of the microglia [66] were obtained at the expense of synaptotoxicity in our model system.

So far, the signal transduction cascades and molecular mechanisms promoting specific functional microglia states are less understood. Overall, the quality and quantity of a neuroinflammatory response are orchestrated by the action of different cytokines and other factors, which are able to trigger selective genetic programs in microglia and also astrocytes and thereby adjusting their functional capabilities. Secretions of pro-inflammatory cytokines like TNFα, IL-1β, and IFNγ favor the polarization of microglia into the classical M1-like direction, leading to the generation of ROS, impairment of neuronal functions, and promoting neurodegeneration. In contrast, the secretion of cytokines such as IL4, IL10, or TGFβ promotes the protection of neurons by clearing cellular debris and toxins like Aβ via stimulating microglial M2-like polarization [5,16,19]. Microglial clearance of Aβ is mainly performed by a phagocytic-proficient M2-like population, which is characterized by the expression of the typical markers such as Arg-1, MRC1, Ym-1, and FIZZ1 [5,16,19]. In favor of improved clearance of Aβ, these classical marker genes were found to be significantly upregulated in the cortex and hippocampus of Ttg mice compared to APP23 animals, and importantly, they were selectively elevated in isolated microglia derived from mice with astrocytic IKK2/NF-κB activation. Furthermore, we also detected the high expression of Gpnmb, a novel M2-like marker gene of microglia, which belongs to the group of Alzheimer’s disease-associated marker genes [67]. During AD pathology, microglia can switch from a homeostatic to a disease-associated phenotype, which has been called “microglial neurodegenerative phenotype” (MGnD) or “disease-associated microglia” (DAMs), and which is characterized by the upregulation of a subset of genes including Gpnmb, ApoE, Trem2, and others [17,27]. Taken together, our data show that astrocytic IKK2/NF-κB activation is sufficient to initiate a microglial activation program characterized by an M2-like gene expression profile in microglia which is associated with efficient phagocytosis and Aβ uptake. In support of this, mixed glial cultures where IKK2/NF-κB signaling was directly activated in microglia cells exhibited a gene expression pattern indicative for M1-like or LPS-induced-like polarization with downregulation of M2-like marker genes (Figure 9). However, in future studies, it will be important to use our model systems to directly determine the ex vivo phagocytic activity of the different microglia populations.

AD is a highly heterogeneous disease, and the role of non-neuronal cells in AD onset and progression is not fully elucidated. Beyond the potential paracrine effects on microglia activation, IKK2/NF-κB-mediated astrocyte activation may also directly affect Aβ homeostasis. As C3 is also highly upregulated in astrocytes, astrocyte-regulated phagocytosis via interaction with the CR3 receptor (also known as Mac-1) might participate in phenotype development in our model. Transcriptomic studies with astrocytes from AD mouse models and the human AD brain support the idea that activated astrocytes in AD may lose their neuroprotective function [68,69]. In line with this, we observed a significant downregulation of the glutamate transports EAAT1 and EAAT2 in isolated astrocytes with activated NF-κB suggesting that these astrocytes lose their ability to prevent excitotoxicity (data not shown). However, further analyses are necessary to determine whether astrocytic IKK2/NF-κB activation triggers a similar gene expression program recently found in so-called disease-associated-astrocytes (DAA) in AD, which appeared at early disease stages and increased in abundance with disease progression [70].

To exclude any mutual transgene interference accounting for the loss of Aβ in our experimental system, we determined overall APP^SWE^ synthesis by Western blotting using soluble and insoluble protein fractions and detected a reduction of 50% in Ttg mice compared to APP23 controls. Upon β-secretase-mediated (BACE1) cleavage of the β C-terminal fragment (β-CTF/C99) from APP, the γ-secretase complex produces the Aβ peptides associated with AD. Importantly, β-CTF has been shown to accumulate before Aβ peptides aggregate in vivo and, therefore, it is proposed to be an early and critical factor for initiating neurodegenerative processes and cognitive decline in AD [71,72,73]. Our data demonstrate strongly reduced levels of both β-CTF/C99 and Aβ monomers in insoluble protein fractions from Ttg mice (using 6E10 antibody) supporting the beneficial outcome on plaque burden upon astrocytic IKK2/NF-κB activation.

The aggregation of Aβ and amyloid formation has been described as a nucleation-dependent polymerization process, including an initial slow nucleation phase, also called lag-phase, followed by a rapid growth phase resulting in plaque formation [74]. The addition of stable seeds, e.g., performed aggregates from different sources including human post-mortem brain tissue obtained from AD patients can accelerate nucleation and significantly shorten the lag phase in a process termed “seeding” [75,76]. Interestingly, we observed a prominent reduction in plaque number, whereas overall plaque size was less affected in our model. This suggests that the specific microglia population in Ttg mice may prevent the initial nucleation process, e.g., by dampening the critical concentration of monomeric Aβ needed for polymerization or by eliminating emerging micro plaques. In contrast, once bigger plaques have formed, Ttg microglia are less qualified to inhibit further plaque growth. Moreover, we showed that early microglia differentiation and expansion, triggered by precocious activation of IKK2-NF-κB in astrocytes, might be responsible for inducing a beneficial phenotype that persists at later stages of the disease, where AD pathology is advanced. The precocious activated phenotype and consequent expansion/proliferation in the early phase of AD may guarantee a better efficacy in clearing and phagocytosis of the plaques. Indeed, studies have demonstrated that in the early stages of AD, the microglial function is neuroprotective, acting to clear apoptotic cells and pathological protein aggregates [77] as well as forming a barrier around plaques to restrict their growth and diffusion of synaptotoxic Aβ oligomers [78,79]. On the other hand, under aging conditions, Aβ oligomers can trigger a potent inflammatory response in microglial cells through NF-κB and JNK signaling [80] leading to an M1-like phenotype. This impairs microglial phagocytosis and clearance of Aβ fibrils, thereby contributing to an initial neurodegenerative evolution of AD.

In order to characterize the underlying cellular and molecular mechanisms reducing the plaque burden in Ttg mice, we also measured the mRNA expression of genes encoding proteins crucially involved in APP processing (e.g., BACE1 and others). We could not detect obvious changes in Ttg mice compared to APP23 controls that could give an immediate explanation for the prominent reduction in β-CTF and Aβ. However, we detected a 2–3-fold increase in ADAM17 and BACE2 gene expression only in the hippocampus, a constellation possibly supporting the non-amyloidogenic processing of APP [23,81,82] which may account for the reduced amyloid plaque load in the hippocampus of Ttg animals but does not hold true for the cortical area. We also tested other factors, such as MMPs known to be involved in the intra- and extra-cellular degradation of β-CTF and Aβ, and found them only marginally affected in Ttg mice. Although higher expression of MMP2 might contribute to the reduction in plaque load in Ttg mice, this might be counterbalanced by the downregulation of IDE. As no significant MMP2 induction is observed in the cortex, where the plaque burden was also reduced in the Ttg animals, the hippocampal upregulation of MMP2 is unlikely to account for the overall reduction of amyloid deposition in the brain. From these results, we cannot exclude that other, alternative APP processing mechanisms are involved in phenotype development [83].

However, we propose that Aβ production per se is not impaired to a great extent in our Ttg AD model system. In fact, cellular and molecular mechanisms responsible for improved clearance of Aβ peptides are much more plausible processes, which can be influenced in a positive manner by the specific innate immune response induced by astrocytic IKK2/NF-κB activation. Vice versa and supporting this view, deficits in inflammation-associated processes involved in Aβ clearance are increasingly recognized as crucial events in AD pathogenesis [47,48,60,84,85]. Our study highlights novel aspects of inflammatory responses by astrocytes and microglia associated with neurodegeneration in general and in the context of AD. Although AD etiology has not been completely established, multiple lines of evidence indicate that neuroinflammation has emerged as an important component of AD pathology. There is also increasing evidence for the presence of pathogens in the brains of AD patients; therefore, it is reasonable to mention that viral, bacterial, and fungal infections might be causative factors for the inflammatory pathway in AD and as a consequence represents a potential cause of AD [86,87,88]. As it is well known that extracellular stimuli for NF-κB include various bacterial and viral products, it is essential to obtain a detailed understanding of the innate immune response in AD pathogenesis to differentiate the protective arm of neuroinflammation from the detrimental one.

## 5. Conclusions

Our findings implicate that specific activation of the innate immune system in the brain, in particular, IKK2/NF-κB-dependent reactive astrogliosis is able to orchestrate a neuroinflammatory response which improves the plaque clearance capabilities of microglia cells and/or promotes a shift toward the non-amyloidogenic APP processing pathway in the APP23 model. In this scenario, astrocyte-derived, paracrine-acting factors, which allow selective modulation of microglia function to a phagocytosis-proficient state would be interesting targets for therapeutical intervention. In addition, extrinsic factors able to modulate APP processing would be an attractive therapeutical strategy. For this, it is important to clarify in the future whether paracrine factors derived from astrocytes are acting in our model system.

## Figures and Tables

**Table 1:** Gene regulation of selected markers in primary microglial cells.

Microglia	In Vivo			Ex Vivo		
Gene Name	Mean Gene Expression		Significance	Mean Gene Expression		Significance
(a) Polarization-associated	Control (Co)	IKK2-CA		EtOH	4-OHT	
*Cd14*	4.859	7.054	ns	37.68	80.44	*
*Cd86*	11.22	13.46	ns	4.213	1.589	**
*Cd163*	0.9658	0.3654	**	0.02	0.0866	*
*Inos*	0.06810	0.05243	ns	na	na	na
*Mrc1*	1.075	2.323	ns	8.459	0.647	*
*Ym1*	0.9075	15.39	**	nd	nd	nd
(b) Inflammation-associated	Control (Co)	IKK2-CA		EtOH	4-OHT	
*C3*	2.893	29.29	***	2.15	155.26	**
*Ccl2*	1.287	3.525	**	0.6	3.32	*
*Ccl5*	0.1399	0.867	*	0.05	5.19	**
*Ccl6*	17.96	36.8633	ns	44.95	5.992	****
*Gpnmb*	0.1434	0.8237	**	3.436	1.156	ns
*Gbp2*	0.6915	3.618	***	0.09	3.28	***
*Il1b*	7.363	26.35	*	1.22	92.112	*
*Lcn2*	4.681	1.710	*	0.06	262.59	*
*Spp1*	6.622	11.75	ns	111.4	27.02	**
*Tgfb*	73.22	67.22	ns	53.25	52.49	ns
*Tgm1*	0.1797	0.5212	**	0.073	2.808	**
*Tnfa*	1.22	3.037	*	2.134	9.581	***

The different types of microglial cells are derived from one-year-old IKK2-CA^GFAP^ mice with astrocytic IKK2/NF-κB activation (in vivo situation) and from mixed glial cultures derived from the CX3CR1-CreERT2/CAG-LSL-IKK2-CA model with direct IKK2/NF-κB activation (ex vivo condition). The table illustrates the different gene profile of markers related to microglia polarization/differentiation and inflammatory-associated markers expressed by primary cells derived from Co and GFAP/IKK2-CA mice (in vivo) and from EtOH (Co)- and 4-OHT (IKK2-CA)-induced cells (ex vivo). Mean values are shown as qPCR-relative expression vs Hprt, n = 4–6. Expression is normalized to the HPRT housekeeping gene. Statistical analysis: ANOVA multiple comparison test (* *p* = 0.05; ** *p* = 0.01; *** *p* = 0.001; **** *p* = 0.0001; ns = non-significant; na = not analyzed; nd = not detectable).

## Data Availability

Not applicable.

## Supplementary Materials

The following are available online at https://www.mdpi.com/article/10.3390/cells10102669/s1, Figure S1: Functional characterization of the triple-transgenic GFAP.tTA/tetO.IKK2-CA/APP23 (Ttg) mouse model of AD with chronic neuroinflammation. Figure S2: Activation of IKK2/NF-κB signaling in astrocytes induces strong astrogliosis and microgliosis. Figure S3: Astrocytic-driven neuroinflammation in APP23 animal model induces plaque load reduction and decreased number of plaques in the brains of Ttg mice. Figure S4: Activation of constitutive IKK2/NF-κB signaling in astrocytes in the APP23 animal model induces differences in plaque morphology together with changes in local gliosis surrounding the plaques. Figure S5: Expression of enzymes involved in the cleavage of monomeric Aβ and in the degradation of Aβ fibrils. Figure S6: NF-κB activation in astrocytes promotes a neuroinflammatory response characterized by a mild increase in M1-like gene expression.
