# Supplementary material for: IKK2/NF-κB Activation in Astrocytes Reduces amyloid β Deposition: A Process Associated with Specific Microglia Polarization
Source: Cells. 2021 Oct 6;10(10):2669. doi: 10.3390/cells10102669 (PMC8534251; doi:10.3390/cells10102669)
Supplement: Supplementary file 1 [file cells-10-02669-s001.zip › cells-1359168-SI.pdf]

IKK2/NF-κB Activation in Astrocytes Reduces Amyloid β Deposition: A Process Associated with Specific Microglia Polarization

Shu Yang, Alexander Magnutzki, Najwa Ouali Alami, Michael Lattke, Najwa Quali Alami, Tabea Mellissa Hein, Judith Stefanie Scheller, Carsten Kröger, Franz Oswald, Deniz Yilmazer-Hanke, Thomas Wirth and Bernd Baumann

Supplementary Materials:

Figure S1:

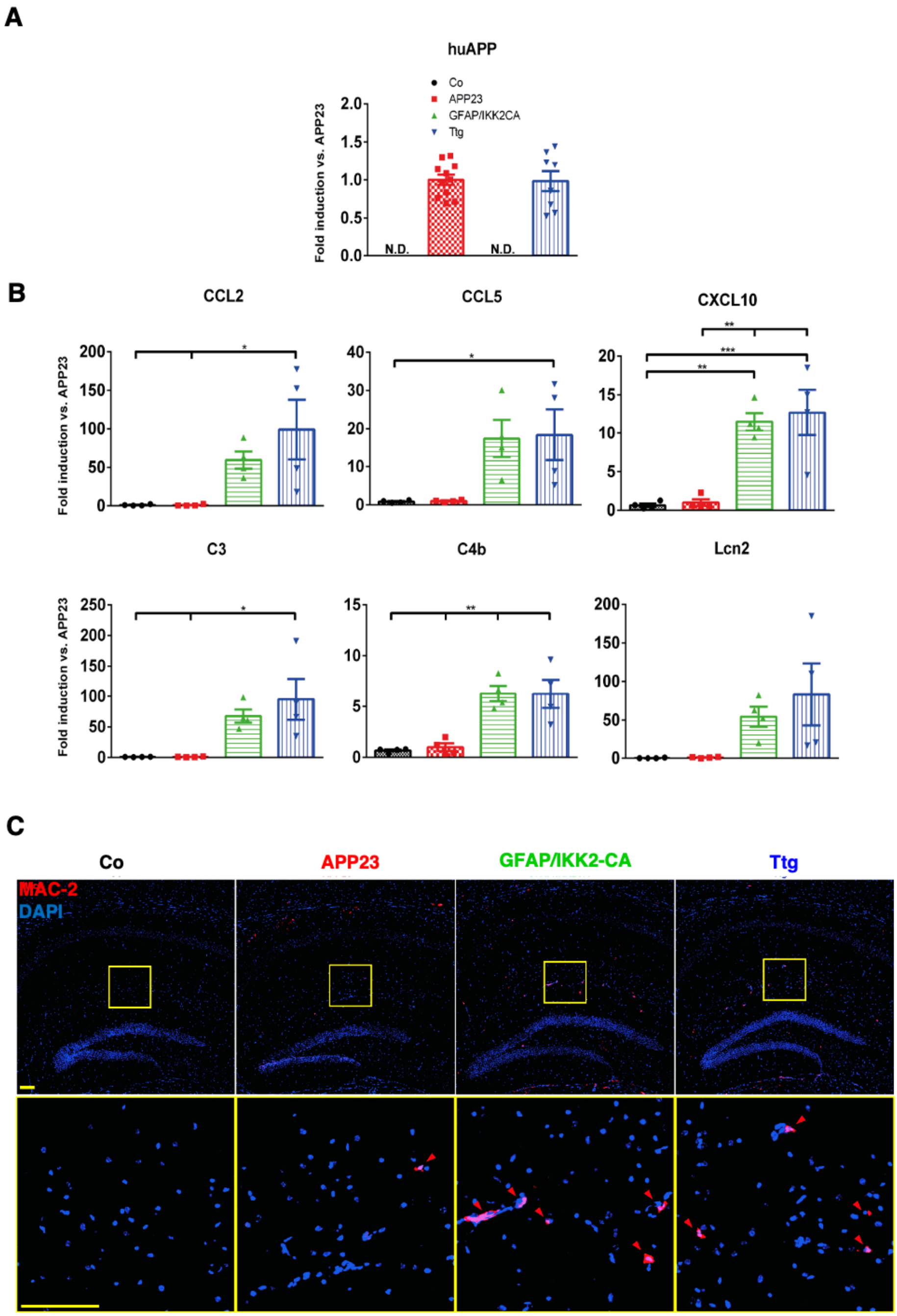

Supplementary Figure S1. Functional characterization of the triple transgenic GFAP.tTA/tetO.IKK2-CA/ APP23 (Ttg) mouse model of AD with chronic neuroinflammation. (**A**) qRT-PCR determination of human APP (huAPP) mRNA levels derived from hippocampal samples of Co, APP23, GFAP/IKK2-CA and Ttg mice at 12-months of age. Expression is normalized to APP23 littermates. (**B**) qRT-PCR determination of mRNA levels of pro-inflammatory chemokines and factors (CCL2, CCL5, CXCL10 and C3, C4b and Lcn2) derived from hippocampal samples of Co, APP23, GFAP/IKK2-CA and Ttg mice at 12-months of age. Expression is normalized to APP23 mice. Results show significant upregulation of all genes in GFAP/IKK2-CA and Ttg mice compared to Co and APP23 littermates as indicated. (**C**) Representative panel depicting IF staining of MAC-2 protein (red), a marker of activated macrophages, in hippocampal paraffin sections (7 µm thickness, Scale bar = 100 µm) of Co, APP23, GFAP/IKK2-CA and Ttg littermates. Nuclei are labeled with DAPI (blue). Yellow squares frame a sub-region located in the molecular layer of the dentate gyrus, which is depicted at higher magnification in the lower part of the panel (Scale bar = 20 µm). Statistical analysis: ANOVA multiple comparison test (**p* = 0.05; ***p* = 0.01; ****p* = 0.001).

Figure S2:

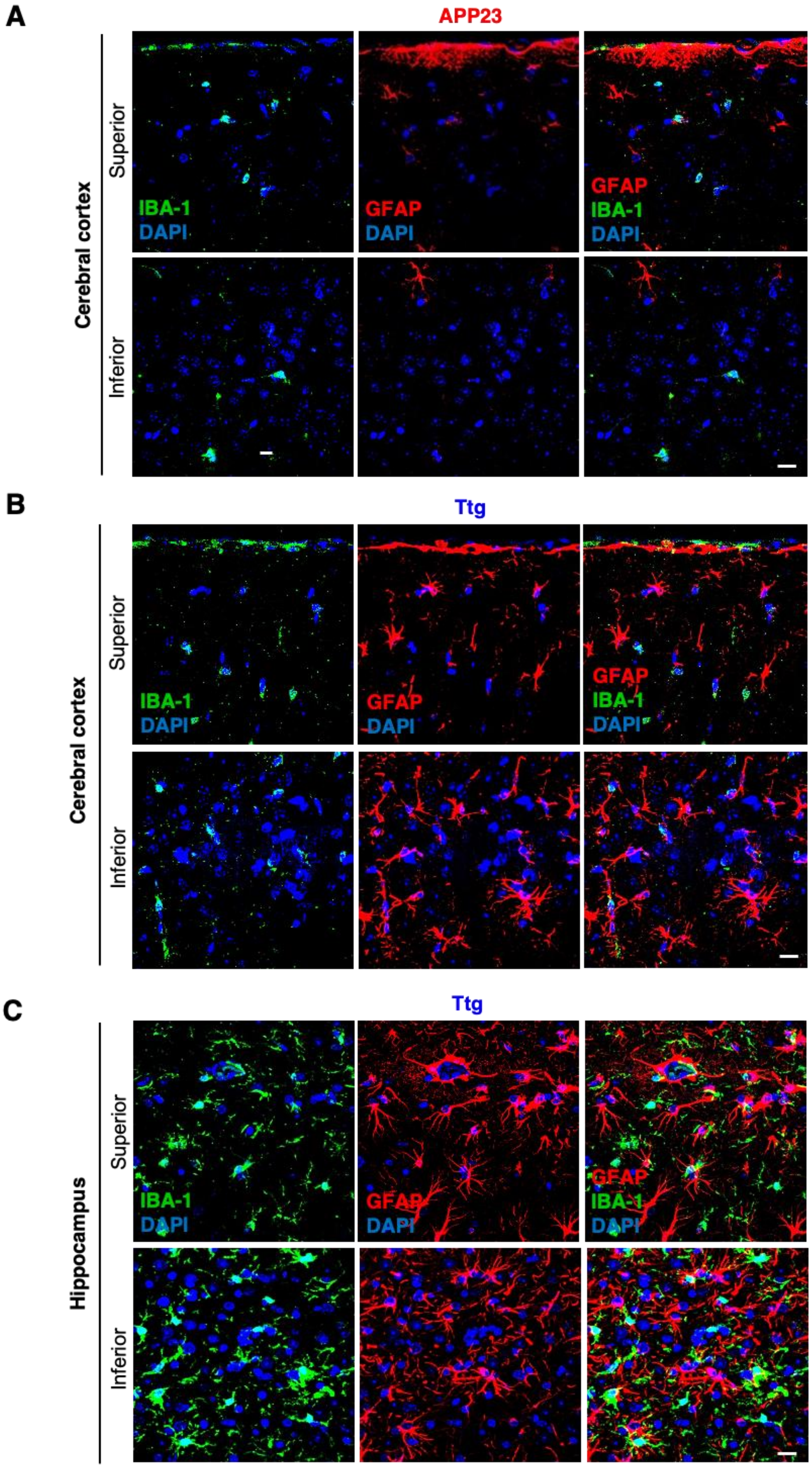

Supplementary Figure S2. Activation of IKK2/NF- B signaling in astrocytes induces strong astrogliosis and microgliosis. (**A**) Representative pictures of paraffin section (7 μm thickness) showing IF staining of microglial cells

marker IBA-1 and astrocytic marker GFAP in the superior and inferior cerebral cortex of APP23 and (**B**) Ttg mice. (**C**) Representative pictures of paraffin section (7 µm thickness) depicting IF staining of microglial cells marker IBA-1 and astrocytic marker GFAP in the superior and inferior hippocampus of Ttg mice. Nuclei are labeled with DAPI (blue). Scale bar = 20 µM.

Figure S3:

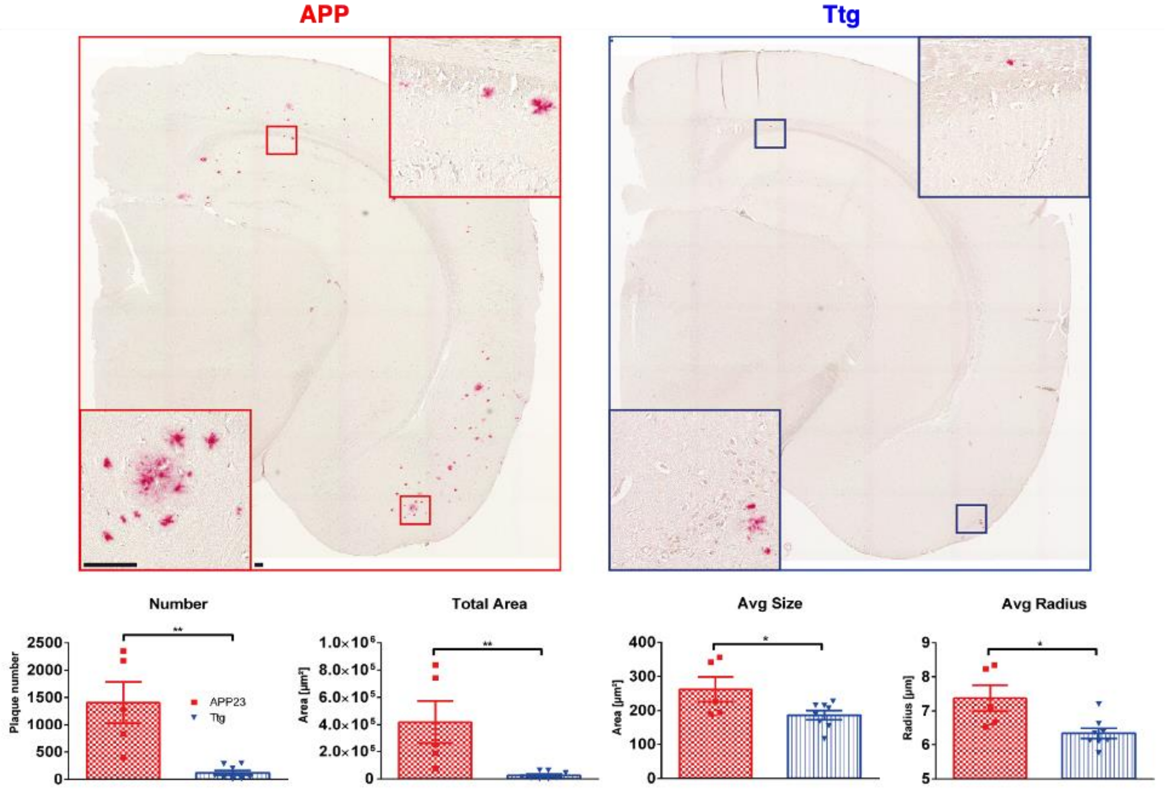

B

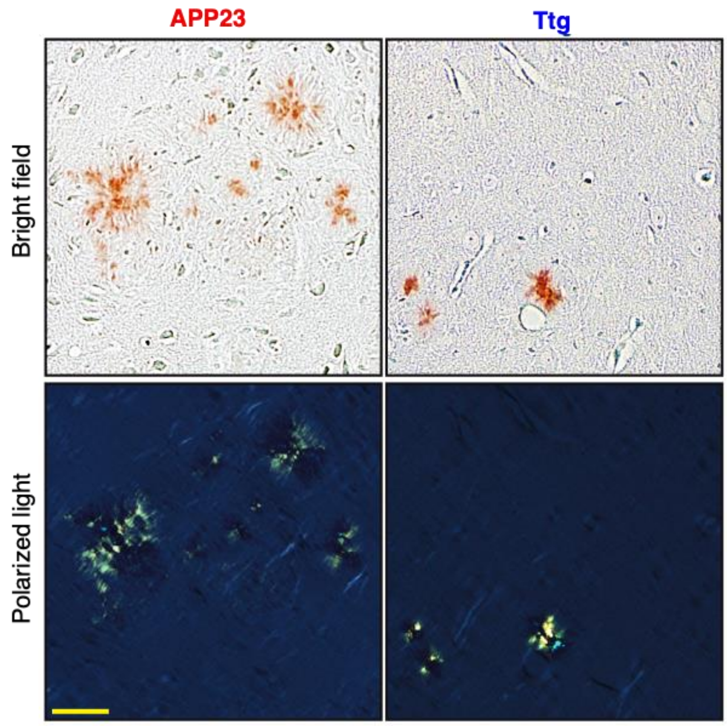

Supplementary Figure S3. Astrocytic driven neuroinflammation in APP23 animal model induces plaque load reduction and decreased number of plaques in the brains of Ttg mice. (**A**) Panel showing Congo red staining of cortical sections from APP23 and Ttg mice. Analysis reveals a pronounced reduction of amyloid plaque (pink) number and size. Bar diagrams show quantification of the number and the total area occupied by plaques in the

whole brain, by evaluation of sequential sections. Average size and radius of remaining plaques is significantly reduced in the Ttg mice compared to APP23 mice. (**B**) Panel showing bright-field pictures (on the top) of Aβ amyloid IHC staining in hippocampal area of APP23 and Ttg mice. Amyloid plaques show characteristic "apple-green" birefringence when visualized in polarized light (in the bottom). Amyloid burden is strongly reduced in Ttg mice compared to APP23 littermates. Statistical analysis: Student's t test (**p* = 0.05; ***p* = 0.01); Scale bar = 100 μm.

Figure S4:

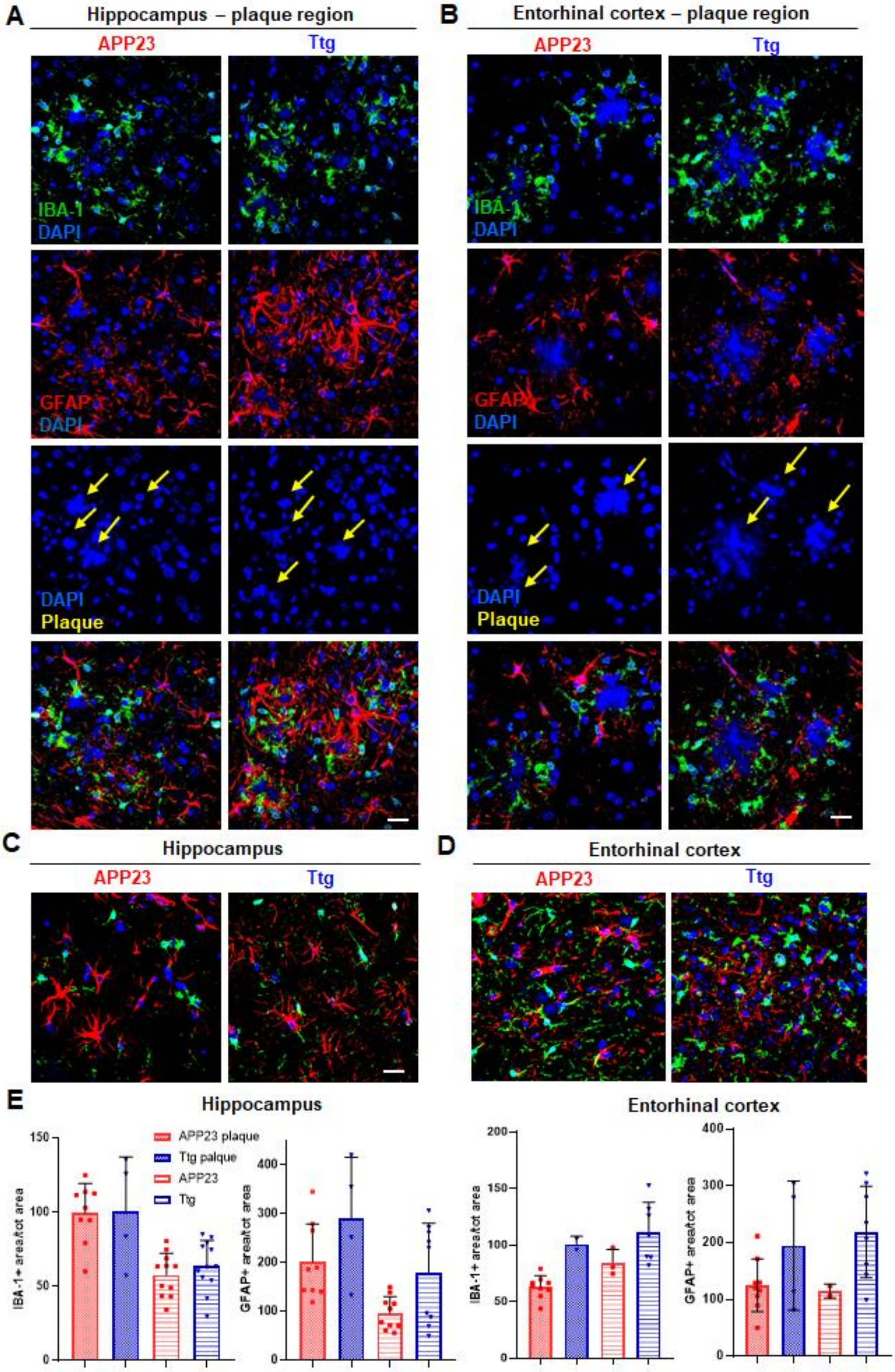

A
Hippocampus – plaque region
APP23
Ttg
IBA-1
DAPI
GFAP
DAPI
DAPI
Plaque
B
Entorhinal cortex – plaque region
APP23
Ttg
IBA-1
DAPI
GFAP
DAPI
DAPI
Plaque
C
Hippocampus
APP23
Ttg
D
Entorhinal cortex
APP23
Ttg
E
Hippocampus
Entorhinal cortex
APP23 plaque
Ttg palque
APP23
Ttg
IBA-1+ area/tot area
GFAP+ area/tot area

Supplementary Figure S4. Activation of constitutive IKK2/NF-κB signaling in astrocytes in the APP23 animal model induces differences in plaque morphology together with changes in local gliosis sur-rounding the plaques. (**A**) Representative panel showing IF staining of microglial cells (IBA-1 in green) and astrocytes (GFAP in red) surrounding the plaques (indicated by yellow arrows), lo-cated in the hippocampal area of APP23 and Ttg mice. (**B**) Representative panel depicting IF staining of IBA-1 positive microglia and GFAP positive astrocytes around the Aβ plaques (indi-cated by yellow arrows), located in the entorhinal cortex of APP23 and Ttg mice. (**C**) Representative picture of microglia (IBA-1 in green) and astrocytes (GFAP in red) located in the hippocampal region far from the Aβ plaques. (**D**) Representative picture of microglia (IBA-1 in green) and as-trocytes (GFAP in red) in the entorhinal cortex far from the Aβ plaques. (**E**) Quantitative analysis of IBA-1 and GFAP positive area in the hippocampus and in the entorhinal cortex. Filled columns indicate IBA-1 and GFAP positive area quantification in regions surrounding the plaques, while striped columns indicate quantification of IBA-1 and GFAP positive area in regions far from the plaques. Nuclei are labeled with DAPI (blue). Scale bar = 20 μm.

Figure S5:

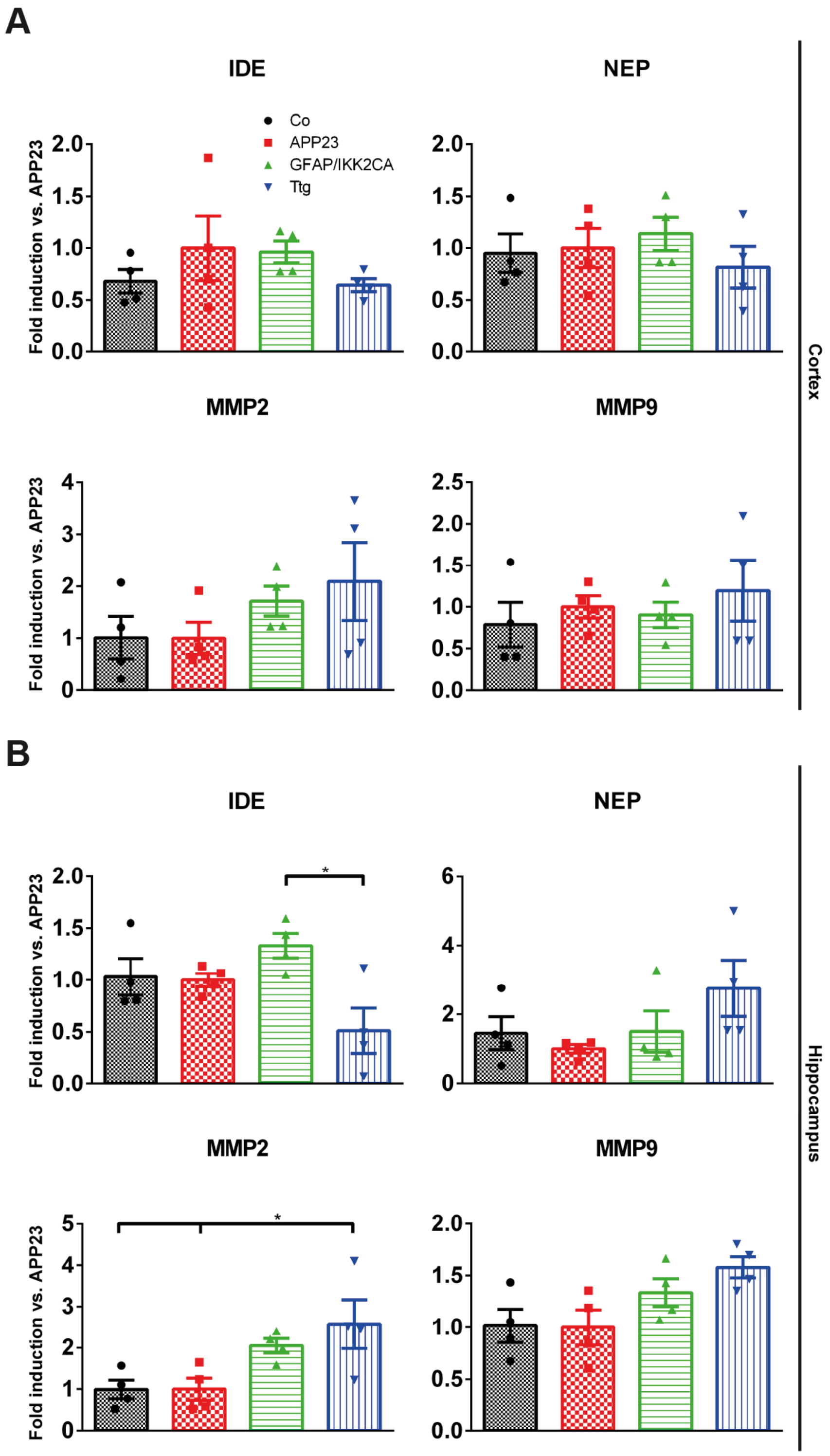

Supplementary Figure S5. Expression of enzymes involved in the cleavage of monomeric Aβ and in the degradation of Aβ fibrils. qRT-PCR analysis of IDE, NEP, MMP2, MMP9 mRNA levels in the cortex (**A**) and hippocampus (**B**) of Co, APP23, GFAP/IKK2-CA and Ttg mice. Quantitative analysis shows a significant reduction in IDE and three-fold increase in MMP2 gene expression in the hippocampal region of Ttg mice compared to APP23 littermates. Expression is normalized to APP23. Statistical analysis: ANOVA multiple comparison test (*p = 0,05). IDE = Insulin degrading enzyme; NEP = Nuclear export protein; MMP2 = Ma-trix Metallopeptidase 2; MMP9 = Matrix Metallopeptidase 9.

Figure S6:

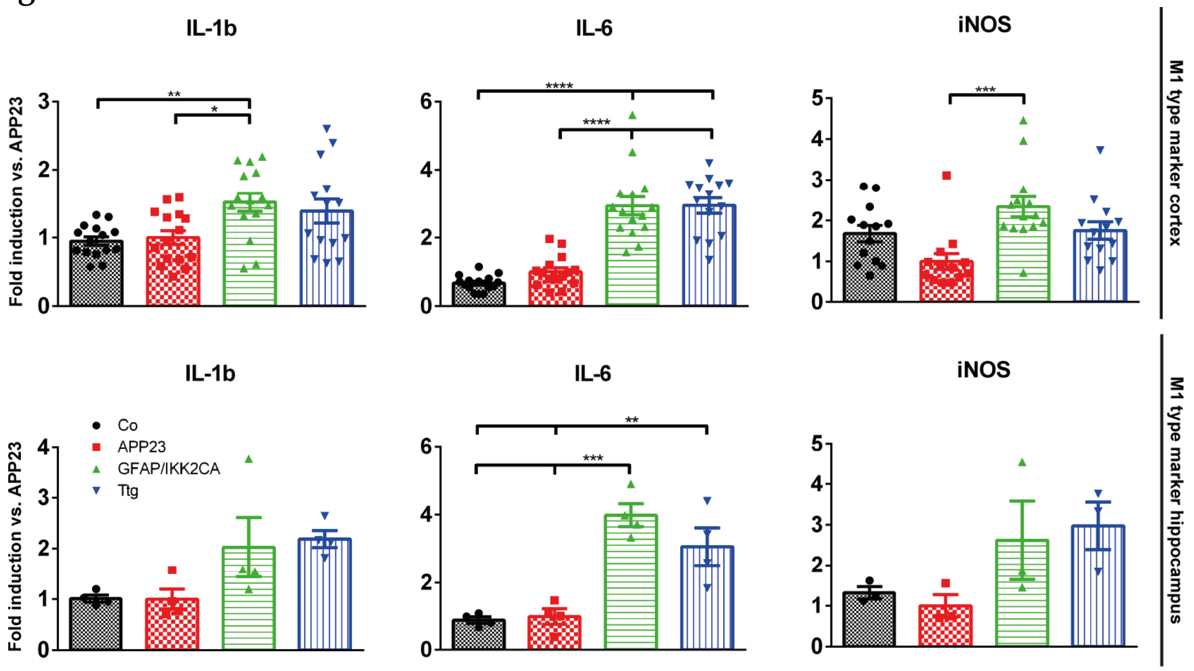

Supplementary Figure S6. NF-κB activation in astrocytes promotes a neuroinflammatory response characterized by a mild increase in M1-like gene expression. qRT-PCR mRNA levels of M1-related markers IL-1b, IL-6 and iNOS in cortical and hippocampal regions of Co, APP23, GFAP/IKK2-CA and Ttg mice as indicated. Expression is normalized to APP23. Il-6 is significantly elevated in both the cortex and hippocampus in GFAP/IKK2-CA and Ttg mice whereas IL-1b and iNOS is only in creased in the cortex of GFAP/IKK2-CA mice. Statistical analysis: ANOVA multiple comparison test (**p* = 0.05; ***p* = 0.01; ****p* = 0.001; *****p* = 0.0001).
